# Identification and validation of a novel panel of *Plasmodium knowlesi* biomarkers of serological exposure

**DOI:** 10.1371/journal.pntd.0006457

**Published:** 2018-06-14

**Authors:** Lou S. Herman, Kimberly Fornace, Jody Phelan, Matthew J. Grigg, Nicholas M. Anstey, Timothy William, Robert W. Moon, Michael J. Blackman, Chris J. Drakeley, Kevin K. A. Tetteh

**Affiliations:** 1 Department Immunology and Infection, London School of Hygiene and Tropical Medicine, London, United Kingdom; 2 Menzies School of Health Research and Charles Darwin University, Darwin, Northern Territory, Australia; 3 Infectious Diseases Society Sabah-Menzies School of Health Research Clinical Research Unit, Kota Kinabalu, Sabah, Malaysia; 4 Clinical Research Centre, Queen Elizabeth Hospital, Kota Kinabalu, Sabah, Malaysia; 5 Jesselton Medical Centre, Kota Kinabalu, Sabah, Malaysia; 6 Malaria Biochemistry Laboratory, The Francis Crick Institute, London, United Kingdom; Walter and Eliza Hall Institute, AUSTRALIA

## Abstract

**Background:**

*Plasmodium knowlesi* is the most common cause of malaria in Malaysian Borneo, with reporting limited to clinical cases presenting to health facilities and scarce data on the true extent of transmission. Serological estimations of transmission have been used with other malaria species to garner information about epidemiological patterns. However, there are a distinct lack of suitable serosurveillance tools for this neglected disease.

**Methodology/Principal findings:**

Using *in silico* tools, we designed and expressed four novel *P*. *knowlesi* protein products to address the distinct lack of suitable serosurveillance tools: *Pk*SERA3 antigens 1 and 2, *Pk*SSP2/TRAP and *Pk*TSERA2 antigen 1. Antibody prevalence to these antigens was determined by ELISA for three time-points post-treatment from a hospital-based clinical treatment trial in Sabah, East Malaysia (n = 97 individuals; 241 total samples for all time points). Higher responses were observed for the *Pk*SERA3 antigen 2 (67%, 65/97) across all time-points (day 0: 36.9% 34/92; day 7: 63.8% 46/72; day 28: 58.4% 45/77) with significant differences between the clinical cases and controls (n = 55, mean plus 3 SD) (day 0 p<0.0001; day 7 p<0.0001; day 28 p<0.0001). Using boosted regression trees, we developed models to classify *P*. *knowlesi* exposure (cross-validated AUC 88.9%; IQR 86.1–91.3%) and identified the most predictive antibody responses.

**Conclusions/Significance:**

The *Pk*SERA3 antigen 2 had the highest relative variable importance in all models. Further validation of these antigens is underway to determine the specificity of these tools in the context of multi-species infections at the population level.

## Author summary

Malaria caused by *Plasmodium knowlesi* is the most common form of the disease in Malaysia. The parasite is transmitted from monkeys to humans via the bite of an infected mosquito, with the resulting *P*. *knowlesi* infection potentially leading to severe symptoms and in some cases, death. Although adult males working close to areas with infected monkeys are at the greatest risk of infection, the true extent of the geographical boundaries of *P*. *knowlesi* transmission is as yet unknown. The ability to measure antibodies to infection is a powerful technique that would help to address this deficit. However, currently available recombinant proteins lack the required specificity for this role. Here, we have developed a panel of recombinant proteins for eventual use as serological tools, strongly supported by robust statistical methods. We envisage that these tools will complement existing approaches to identifying the geographical limits of *P*. *knowlesi* transmission.

## Introduction

*Plasmodium knowlesi* is a simian parasite which can cause zoonotic malaria in humans [1]. Recent evidence suggests that human *P*. *knowlesi* infections are a growing public health threat in South East Asia, particularly in Malaysia [2]. *P*. *knowlesi* has the potential to cause severe disease in endemic regions [3], and is now the most common cause of clinical malaria in Malaysia [4]. *P*. *knowlesi* is morphologically similar to *P*. *malariae* [5], historically leading to the misdiagnosis of *P*. *knowlesi* infections as *P*. *malariae* [6]. Recent publications have also demonstrated misdiagnosis of *P*. *knowlesi* as *P*. *vivax* and *P*. *falciparum* [7, 8] with potential delay of appropriate treatment associated with case fatalities [3, 9, 10]. Studies have shown that antibodies to *Plasmodium* proteins persist for long periods [11], even in the context of limited exposure or absence of infection. Such antibodies can be utilised in serological assays, accurately estimating the incidence and exposure to *Plasmodium* parasites [12, 13].

One key requirement for serological studies is the identification of *Plasmodium* species-specific biomarkers, particularly in regions where multi-species infections are likely to occur. It is important to distinguish between human serological responses to different *Plasmodium* species to improve our understanding of immunity to these infections, as well as define the geographical spread of infection. Such information can also help to evaluate the impact of how control measures targeting a single species might affect the transmission and immunological profile of other co-endemic species. The few recombinant protein reagents that do exist for *P*. *knowlesi* have a high level of sequence homology with orthologues from other *Plasmodium* species and, as such, are not applicable to identifying species-specific antibody responses. For example, PK66 (*Pk*AMA1) [14] and *Pk*SPATR (secreted protein with altered thrombospondin repeat) [15] share 86% and 85% amino acid identity respectively with *P*. *vivax* (https://is.gd/MzISez), potentially making it difficult to distinguish between the two species where infections are co-endemic.

The 2011 WHO consultation panel on the public health importance of *P*. *knowlesi* recommended the urgent development of *P*. *knowlesi*-specific diagnostic tools [16]. Key to achieving this goal would be the development of sensitive and accurate tools to help monitor the transmission of infection.

In this study, we describe the development and evaluation of a panel of novel recombinant antigens based on *P*. *knowlesi*-specific amino acid sequences, using publicly available *in silico* tools. The development of such well-validated species-specific tools represent a potentially important serosurveillance tool to help monitor for historical *P*. *knowlesi* infections in endemic areas. To illustrate how these data can be used to identify seropositive individuals, we utilise data-adaptive statistical methods (boosted regression trees) to classify exposed individuals. By assessing relative variable importance within these models, we identify the antigen responses contributing most to model predictions and potential serological tools for use in epidemiological studies. These reagents will also serve as an important set of tools to help identify correlates of immunity to *P*. *knowlesi*.

## Methods

### Identification and screening of target sequences

**Fig 1:**
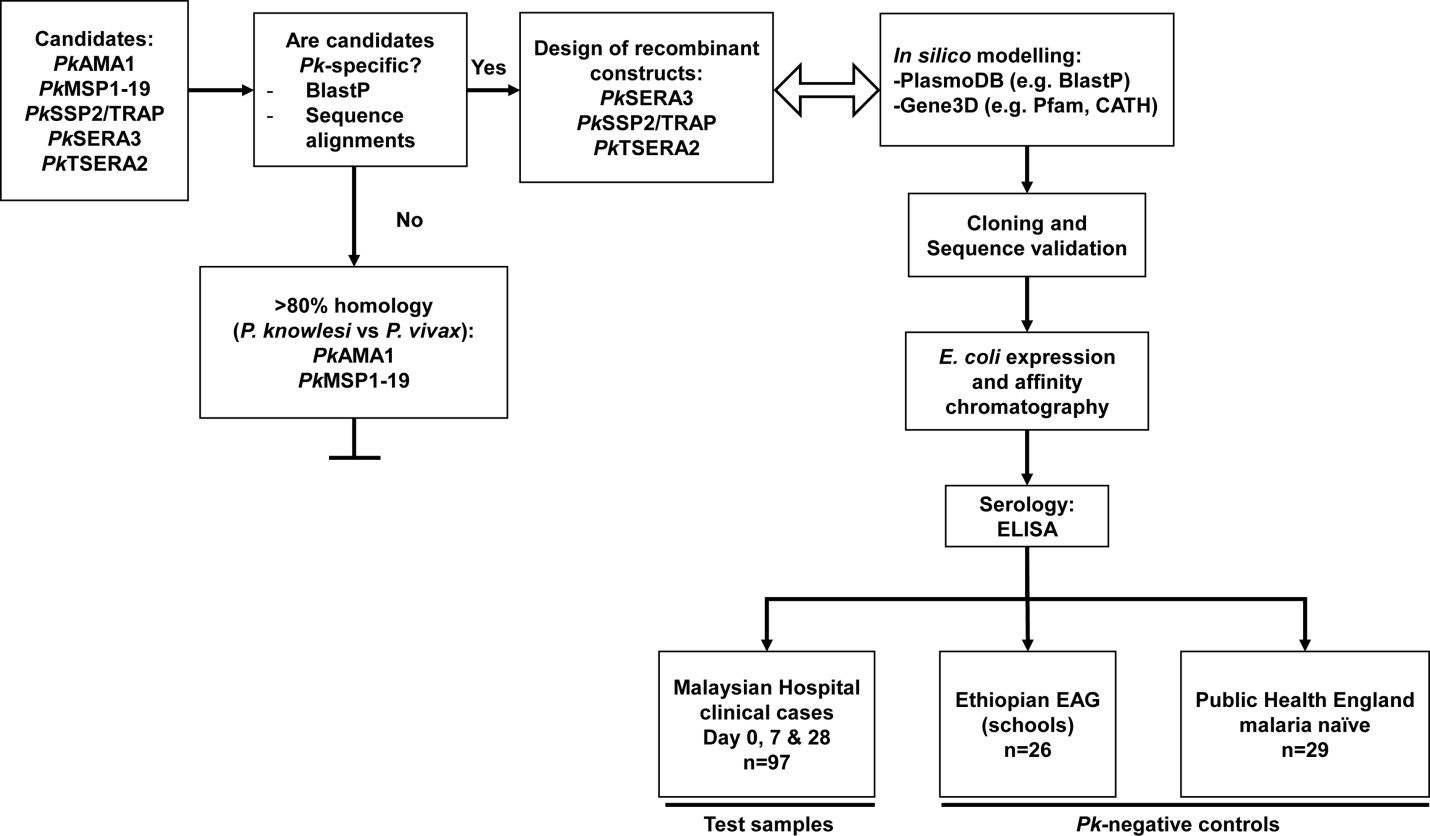
Flowchart summarising the experimental strategy used in the identification and validation of the *P*. *knowlesi*-specific candidates.

Fig 1 outlines the experimental strategy used in the identification of the target sequences of interest. Known markers of seroincidence were selected based on available evidence from *P*. *falciparum*: AMA1 [17], MSP1 [18], SSP2/TRAP [19] and SERA [20] (*Pk*AMA1 (PKNH_0931500), *Pk*MSP1 (PKNH_0728900), *Pk*SSP2/TRAP (PKNH_1265400), SERA3 (PKNH_0413400) and TSERA2 (PKNH_0413500), respectively). Full-length protein sequences for each gene were initially screened using the BlastP search tool (Plasmodb: https://is.gd/XOs7vd [21] and NCBI: https://is.gd/MzISez). Amino acid sequences were used to generate maximum likelihood phylograms to summarise the relatedness of each gene target between species (S1A–S1E Fig). Alignments were also generated for each target using amino acid sequences from other plasmodia matching the query sequence using the MUltiple Sequence Comparison by Log-Expectation (MUSCLE) software (http://www.ebi.ac.uk/Tools/msa/muscle/) [22] (S2A–S2E Fig). Each alignment was then interrogated to identify regions of identity primarily with *P*. *vivax* and *P*. *falciparum* but also with *P*. *malariae* and *P*. *ovale*. Regions or entire sequences showing high levels of identity were excluded from further analysis and the *P*. *knowlesi*-specific truncated regions were again screened using BlastP to validate sequence specificity (Fig 1 and S1 Table). Each target sequence was analysed using domain prediction software (http://gene3d.biochem.ucl.ac.uk/ and http://smart.embl-heidelberg.de/) to help define putative domain boundaries, where possible. The aim was to limit the level of potential antibody cross-reactivity, which would limit the usefulness of the antigens as serological tools due to the high level of identical amino acids between species. A particular problem in co-endemic settings. Simultaneously, sequences were also screened using the TMHMM server (http://www.cbs.dtu.dk/services/TMHMM/) to help confirm the presence, or absence, of signal peptides and transmembrane regions. Previous experience from our group and others has shown that the presence of signal peptides and/or transmembrane domains can significantly impede protein expression and solubility [23]. Based on this, each confirmed target construct was designed to exclude both the signal peptide and transmembrane domains, which in addition to the GST solubility tag was designed to aid expression of soluble proteins [24].

An additional selection criteria step was to determine the transcriptional status of the candidate genes. Blood stage messenger RNA was collected and analysed using the human red blood cell culture adapted *P*. *knowlesi* A1-H1 line [25], grown in human blood obtained from the United Kingdom National Blood Transfusion Service. First strand synthesis was carried out using SuperScript IV Reverse Transcriptase (RT) (Thermo Fisher Scientific) using oligo d(T)20 for priming (RT+) according to the manufacturer’s instructions. As a negative control (RT-), a second identical reaction was set up in parallel without the addition of the SuperScript IV RT. For PCR analysis of cDNA transcripts, RT+ and RT- samples were used as templates for transcript specific PCR primers for the candidate gene sequences alongside genomic DNA controls. In addition, both *Pk*CTRP (circumsporozoite protein and thrombospondin-related adhesive protein [TRAP]-related protein) and *Pk*CSP (circumsporozoite protein), both shown to be pre-erythrocytic stage targets, were included in the panel as negative controls. Where possible, primer pairs were designed to flank introns so that amplicons from cDNA and gDNA could be distinguished. Sequences of primer pairs used to amplify each transcript are listed in S2 Table alongside the expected cDNA and gDNA amplicon size. Amplicons were PCR amplified using GoTaq Green Master Mix (Promega) and analysed on a 1.2% agarose gel (S3 Fig).

### Cloning and expression of *Plasmodium knowlesi*-specific recombinant antigens in *Escherichia coli*

**Fig 2:**
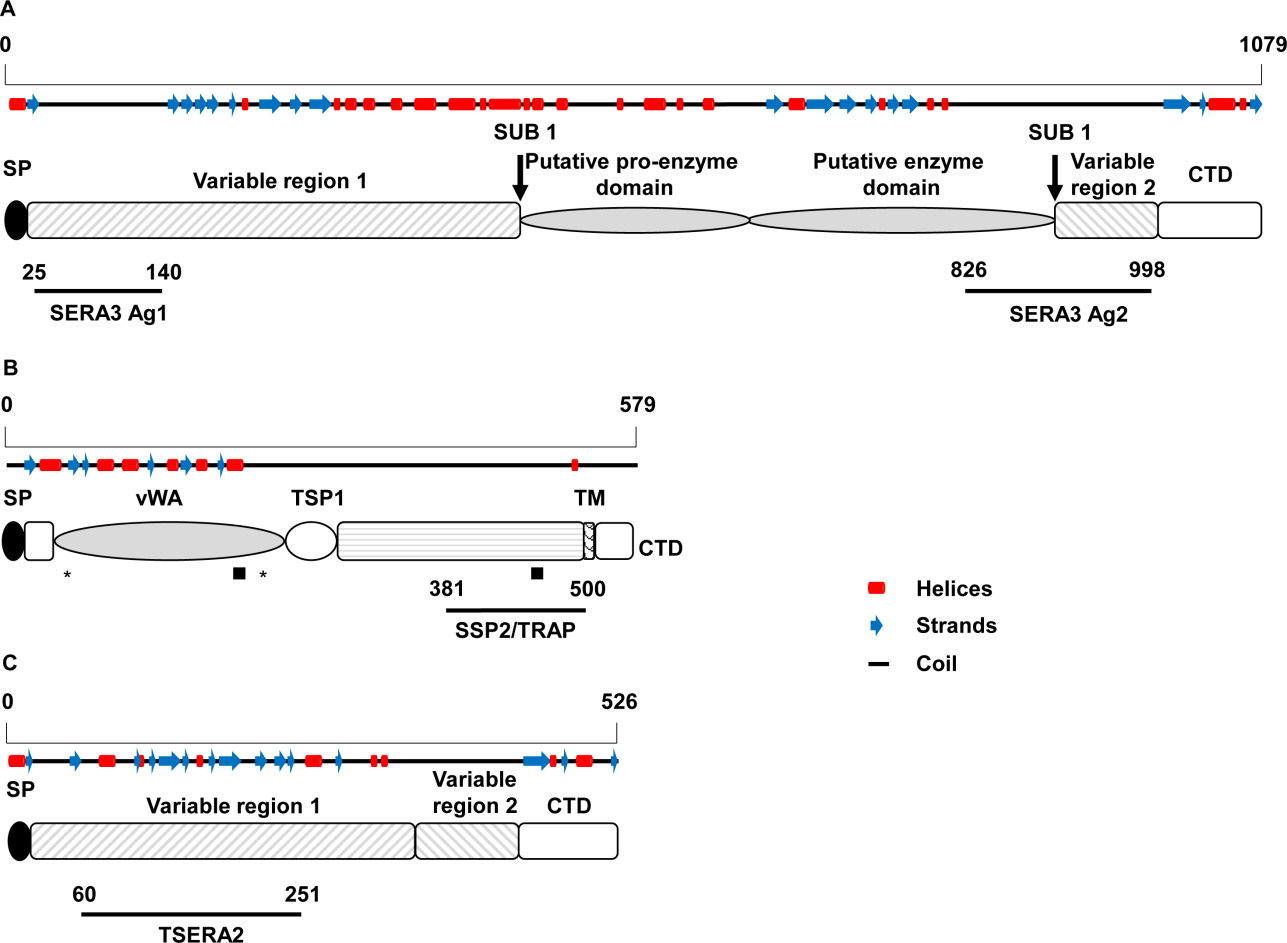
*Plasmodium knowlesi*-specific recombinant antigen constructs. Schematic representations for each protein are shown with key features labelled. (a) *Pk*SERA3 shows the location of the putative pro-enzyme and enzyme domains. The predicted subtilisin (SUB) 1 cleavage sites in relation to variable regions 1 and 2 and the cytoplasmic domain [29]. (b) *Pk*SSP2/TRAP contains a von Willebrandt A domain (vWA), thrombospondin type (TSP) 1 motif, a C-terminal transmembrane (TM) region and a cytoplasmic terminal domain (CTD). Putative T-cell and B-cell epitopes are highlighted with an asterix or black square, respectively [30]. (c) *Pk*TSERA2 shows the lack of central enzyme domain due to truncation of the sequence [29]. Predicted secondary structures generated in I-Tasser [31] are shown above each scheme. Red boxes represent helices, blue arrows sheets and the black line coils. The position of recombinant proteins are highlighted below each scheme with the N- and C-terminal amino acid positions indicated. The overall length of each protein is referenced by the amino acid ruler above each secondary structure prediction. For all proteins SP refers to the signal peptide.

**Fig 3:**
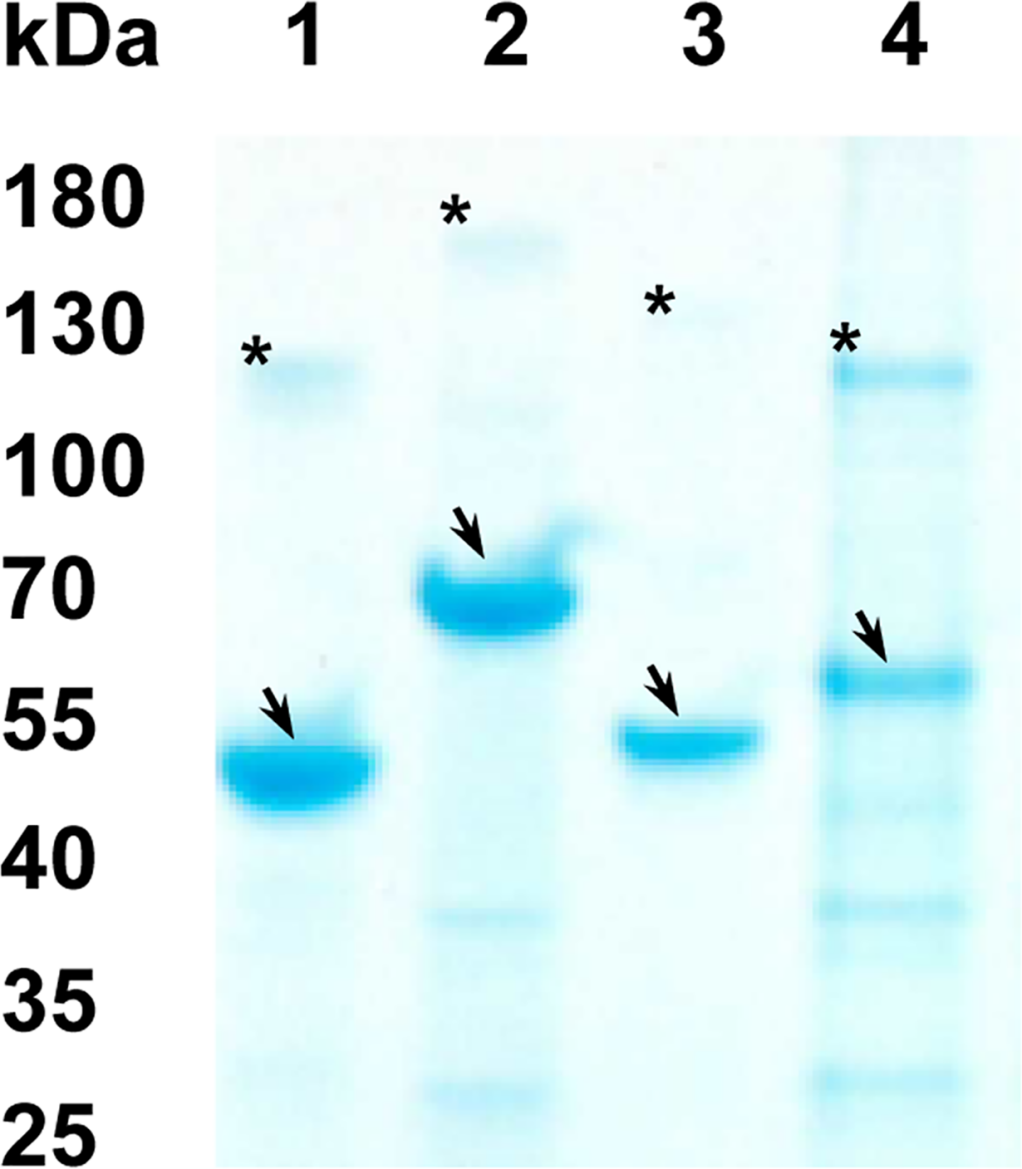
SDS-PAGE of purified recombinants. Lane 1: SERA3 ag1; Lane 2: SERA3 ag2; Lane 3: SSP2/TRAP and Lane 4: TSERA2 ag1. Band sizes are indicated on one side in kDa (Fermentas PageRuler Prestained Protein Ladder). Samples were ran under reducing conditions at approximately 0.4 mg/ml per lane on a 4–20% gradient gel (NuPAGE, BioRad) and stained with Coomassie Blue (BioSafe, BioRad). The arrows and asterisks indicate the protein monomers and aggregates, respectively.

Four new constructs were designed (Table 1 and Fig 2) based on three genes. Two sequences within SERA3 (PKNH_0413400; nucleotide positions 73–419 (Antigen 1) and 2476–2994 (Antigen 2) based on the reference *P*. *knowlesi* H strain), SSP2/TRAP (PKNH_1265400; nucleotide positons 1141–1500) and TSERA2 (PKNH_0413500; nucleotide positons 178–751 (Antigen 1)) and were PCR amplified from *P*. *knowlesi* genomic DNA (H strain). Vector compatible primers were designed for each completed target sequence (S3 Table). Cloning of amplified sequences is as described previously [26]. Briefly, purified inserts were cloned into a TA vector (pGEM-T Easy, Promega) and sequence verified. Correct sequences were restriction digested and sub-cloned into a GST expression vector (pGEX-2T, GE Healthcare) and sequence verified before transforming into BL21 (DE3) *Escherichia coli* expression cells (Novagen). Validated expression clones were expressed automatically using an autoinduction media based on established protocols [27]. Following expression, protein purification was as described [28]. Briefly, GST-tagged proteins from clarified bacterial lysate were purified by affinity chromatography (Glutathione sepharose 4B; GE Healthcare) and fractions from each protein analysed (Bradford assay reagent, BioRad) to identify protein-containing fractions. Pooled protein positive fractions were dialysed against PBS and the protein content quantified (Bicinchoninic acid assay (BCA), Thermo Fisher). The dialysed purified proteins were analysed on a 4–20% gradient gel (NuPAGE Bis-Tris acetate) under denaturing conditions and visualised using the Coomassie blue staining method (BioRad BioSafe, USA) (Fig 3).

**Table 1:** Summary of recombinant antigen construct characteristics.

Gene ID	Antigen	Description	Chromosome	AA position	Expression (mg/L)		Size (kDa)	
							Predicted	Empirical
PKNH_0413400	SERA3 ag1	cysteine protease (Serine repeat-like antigen)	4	25–140	20.5	w/ GST	37.7	49.6 (118.9)
						w/o GST	11.3	n/a
PKNH_0413400	SERA3 ag2	cysteine protease (Serine repeat-like antigen)	4	826–998	15	w/ GST	44.9	68.7 (162.7)
						w/o GST	18.4	n/a
PKNH_1265400	SSP2/TRAP	sporozoite surface protein 2, putative, thrombospondin-related anonymous protein (TRAP)	12	381–500	17	w/ GST	39.7	53.1 (132.5)
						w/o GST	13.2	n/a
PKNH_0413500	TSERA2 ag1	Truncated cysteine protease	4	60–251	11.9	w/ GST	46.8	59.7 (117.9)
						w/o GST	20.4	n/a

The empirical sizes of each protein were calculated using the ImageLab (BioRad) software with the PageRuler prestained marker (Fermentas) as a reference standard (Table 1).

### SNP and phylogenetic analysis

Full-length sequence data from Plasmodb and construct-specific truncated sequences generated in-house using Sanger sequencing were mapped to an in-house reference sequence strain (*Pk*-H strain) using the Burrows-Wheeler Aligner (BWA) software (v0.7.5a-r405) [32]. Single nucleotide polymorphisms (SNPs) (S4–S8 Tables) were called using the SAMtools (v1.3) (Sequence Alignment/Map) software using default settings [33] and were filtered to increase stringency and target only high quality variants (missingness<10%, mixed calls<10%). Custom Perl scripts identified overlap between these SNPs and each gene candidate. Variants were annotated using snpEFF (v4.3i) (http://snpeff.sourceforge.net/*)* [34] to retrieve the amino acid position and type effect of the variant. Maximum likelihood phylogenetic trees were constructed from protein sequences using RAxML [35] with a fixed empirical substitution matrix and 200 bootstraps and was visualised using iTOL (http://itol.embl.de) [36].

### Enzyme-linked immunosorbent assay (ELISA) and sera collection

The indirect enzyme-linked immunosorbent assay was performed to screen for antibodies to *P*. *falciparum*, *P*. *vivax* and *P*. *knowlesi* antigens using previously described methods [37]. Briefly, antigens were coated at 50 ng/well and serum samples (diluted from frozen serum stocks) assayed at 1/1000 dilution for both the *P*. *knowlesi* recombinants and the *Pv*MSP1-19 (donated as a kind gift from Tony Holder) positive control antigen. Polyclonal rabbit anti-human IgG-HRP (Dako, Denmark) was used at 1/15,000 dilution and plates were developed using TMB (One component HRP microwell substrate, Tebu-bio). All assays were performed in duplicate. Negative and positive controls, including blank (buffer only) wells were used to help standardise across assay runs. Values in excess of 1.5 CV between duplicates were considered fails and re-ran.

**Fig 4:**
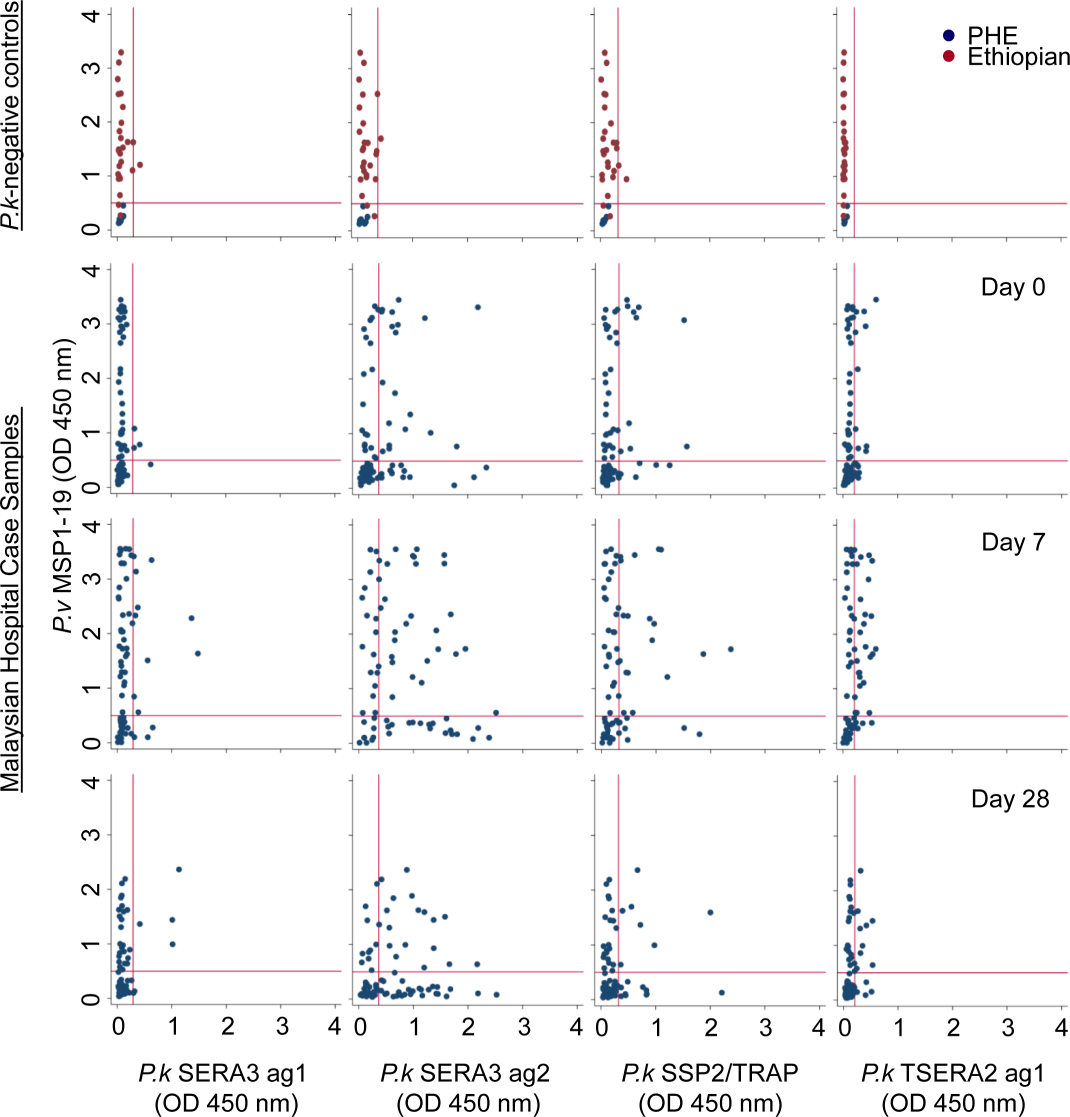
Endemic and *P*. *knowlesi*-negative sera reactivity to *Plasmodium knowlesi-*specific antigens. Scatter plots showing sera reactivity to: *P*. *vivax* MSP1-19 with *P*. *knowlesi* SERA3 ag1 (column 1), SERA3 ag2 (column 2), SSP2/TRAP (column 3) and TSERA2 ag1 (column 4) antigens. Sera samples from *P*. *knowlesi*-negative controls n = 55 (row 1; PHE UK malaria naïve (blue), Ethiopian children (red)) and Malaysian hospital case sera samples from days 0 (n = 92), 7 (n = 72) and 28 (n = 77) of diagnosis (rows 2–4, respectively). The red line in each graph represent the cut off values for the respective *P*. *knowlesi* antigen and was calculated based on Public Health England negative control sera samples (average ODs ± (3xSD)): The vertical cut off line is based on *Pv*MSP1-19 = 0.501. The horizontal cut off line for the *P*. *knowlesi* antigens were based on the following values: SERA3 ag1 = 0.292; SERA3 ag2 = 0.366; SSP2/TRAP = 0.322 and TSERA2 ag1 = 0.208.

Written informed consent was obtained from all study participants [18, 38]. Samples were collected as part of a hospital-based clinical trial in Malaysia, Sabah (www.clinicaltrials.gov: #NCT01708876) (Fig 1) [38]. Serum samples were collected at Day 0 (n = 92), 7 (n = 72) and 28 (n = 77) following hospital admission, with drug treatment also taking place at Day 0. The human research ethics committees of Malaysia (MREC) (#NMRR-12-537-12568), the Menzies School of Health and Research (Australia) (#HREC-2012-1814) and the London School of Hygiene and Tropical Medicine (UK) (#6244) approved the study. Twenty-six *P*. *vivax*-positive Ethiopian samples [18] based on positive responses to *Pv*AMA1 and *Pv*MSP1-19 were used as the *P*. *vivax*-positive, *P*. *knowlesi*-negative control group. In addition, 29 malaria naïve (Public Health England; LSHTM ethics approval #11684) serum samples were used as the *P*. *knowlesi*-negative control group. For the scatterplot presented in Fig 4, both negative control groups were compared to the responses from the *P*. *knowlesi*-exposed hospital clinical case samples. All samples used in the study were anonymised.

### Statistical and sequence analysis

**Fig 5:**
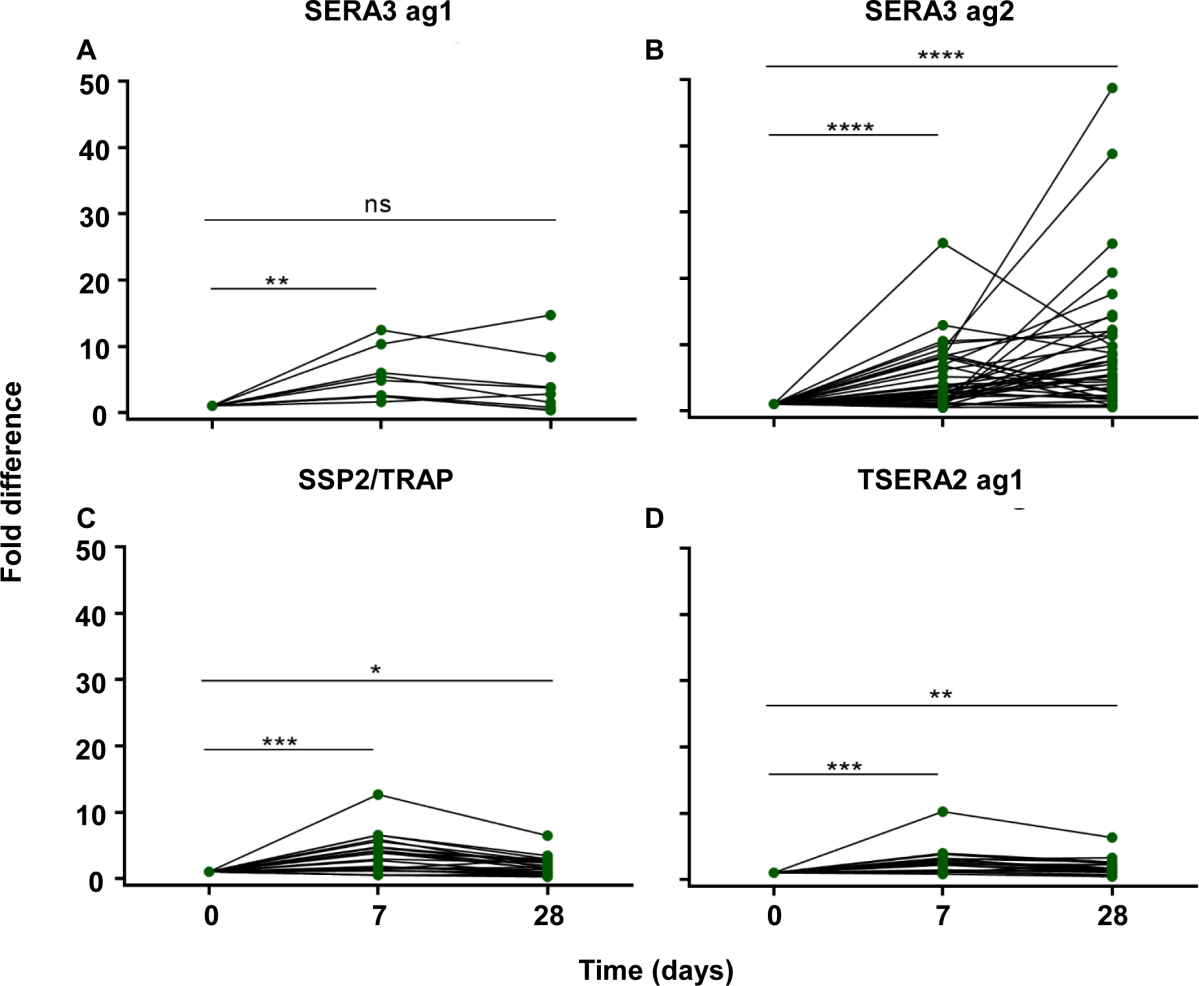
Serial fold increase in antibody reactivity for each antigen following treatment of knowlesi malaria. (a) SERA3 ag1, (b) SERA3 ag2, (c) SSP2/TRAP and (d) TSERA2 ag1. Asterisks indicate level of significance, ns denotes non-significant values (p≤0.0001 ****; p≤0.001 ***; p≤0.01 **; p≤0.05 * and p>0.05 ns).

Descriptive analysis of serological data was performed using STATA/IC 14.2 (StataCorp LP, USA) and PRISM (GraphPad PRISM 7). P values were generated using the Wilcoxon signed rank and Wilcoxon-Mann Whitney tests (STATA/IC 14.2). Scatter plots showing reactivity between *P*. *knowlesi* recombinant antigens and *P*. *vivax* MSP1-19 were created using STATA (Fig 4) and dot plots showing reactivity to *P*. *knowlesi* recombinant antigens were created using GraphPad PRISM (Fig 5 and S4 Fig). Final optical density (OD) values were obtained by subtracting blank OD values, reducing background reactivity. Cut off values for each *P*. *knowlesi*-specific antigen were calculated based on the average ODs of Public Health England negative control sera samples ± (3xSD).

Ensemble boosted regression trees were fit to determine predictive power of antibody responses for classification of *P*. *knowlesi* exposure. To quantify uncertainty around estimates, 100 datasets were assembled including all seronegative individuals from the malaria unexposed population and an equal number of randomly selected *P*. *knowlesi* seropositive individuals (from all time points). All models were fit using stratified 10-fold cross validation with model predictive ability assessed by the area under the receiver operating curve (AUC). The learning rate was set at 0.001 and tree complexity set at 4, to allow for interactions within the dataset. Contribution of responses to each antigen to models was assessed using relative variable importance as described by Elith *et*. *al*.[39]. In this method, the relative importance of individual predictor variables is calculated as the number of times a variable is selected for splitting, weighted by the squared improvement to the model and averaged over all trees and scaled to 100%. Boosted regression tree analysis was completed in R statistical software (v 3.4.2) using the gbm package.

Amino acid sequence alignments were generated using MUltiple Sequence Comparison by Log-Expectation (MUSCLE) (http://www.ebi.ac.uk/Tools/msa/muscle/) [22].

## Results

### *In silico* identification, design and expression of target sequences

Sequences associated with known immunological markers in *P*. *falciparum* were selected based on existing evidence (AMA1 [17, 40], MSP1 [40, 41], SSP2/TRAP [42] and SERA antigens [20, 43]), by interrogating existing *P*. *knowlesi* databases[21, 44] and supporting literature [45] (Fig 1). AMA1 is expressed in the micronemes of both the merozoite (invasive asexual blood stage) and sporozoite (invasive pre-erythrocytive stage) forms [17]. MSP1 is a major protein located on the surface of the merozoite[41]. SSP2/TRAP is also expressed on the surface of the sporozoite forms [42], and the SERA antigens are soluble parasitophorous vacuole proteins [20, 43]. Each sequence was processed using available *in silico* analytical tools (Fig 1). Gene3D [46] and SMART (http://smart.embl-heidelberg.de/) were used to obtain domain prediction information for each gene which helped with the design of truncated fragments (Fig 2). This approach ensured that the design of truncated sequences properly accounted for the presence of any potential domains within each sequence, avoiding unintended truncation of domains which could impact on the solubility of the recombinant proteins. To ensure that expressed products would be specific for *P*. *knowlesi*, target sequences were interrogated multiple times using the BlastP algorithm [47] against both the *Plasmodium* specific (Plasmodb: https://is.gd/XOs7vd [21]) and non-redundant databases (NCBI: https://is.gd/MzISez).

Maximum likelihood phylogenetic trees were constructed using the *P*. *knowlesi* H reference strain, highlighting the relationship of each gene between *Plasmodium* species (S1A–S1E Fig). Specifically, for both *Pv*AMA1 (bootstrap value: 100%) and *Pv*MSP1-19 (bootstrap value: 87%), there is a strong relationship between different *Plasmodium* species, particularly between *P*. *knowlesi* and *P*. *vivax* (S1A Fig), highlighted further by corresponding near identical amino acid alignments (S2A Fig). Amino acid alignments were generated using available sequences for human-pathogenic *Plasmodium* spp., which clearly highlight the level of sequence identity for both genes between *P*. *knowlesi* and *P*. *vivax* (S2A–S2E Fig). Although the bootstrap value strongly supports the grouping of *P*. *knowlesi* with *P*. *vivax* and *P*. *simiovale (P*. *simiovale* was used when data for *P*. *ovale* was lacking) (S2C–S2E Fig; bootstrap value: 100%), the alignments for SSP2/TRAP and the SERA antigens (PKNH_0413400 and PKNH_0413500), help identify regions specific for *P*. *knowlesi* (S2C–S2E Fig). Based on these screens, any sequences showing high amino acid sequence identity to other *Plasmodium* spp., specifically *P*. *ovale*, *P*. *malariae*, *P*. *falciparum* and *P*. *vivax*, were re-edited to focus on *P*. *knowlesi*-specific regions only, where possible. All the antigens were expressed in *Escherichia coli* as soluble products with final yields ranging from 11.9–20.5 mg/L (Fig 3, Table 1).

Based on their predicted molecular masses (including the GST tag), SDS PAGE analysis of the purified proteins clearly suggested multimerisation of the purified products (both monomer and dimer) (Fig 3 and Table 1). The Coomassie stained profiles also illustrated that there is very little non-specific degradation of the recombinant proteins (Fig 3), suggesting that the proteins are stable under the conditions used. The protein sizes for each protein were larger than predicted, so called “gel shifting” when ran on SDS PAGE, which is not uncommon. All though not fully explained for all proteins classes evidence suggests that the presence of acidic residues, net hydropathy or protein aggregation can reduce the effectiveness of SDS in altering the charge, and therefore the migration of proteins through the gel [48, 49]. The fact that all four protein constructs exhibited signs of protein aggregation supports the suggestion that aggregation may affect protein migration on polyacrylamide gels (Fig 3 and Table 1). By way of further validation each protein construct was sequence verified to confirm each sequence and the position of the stop codons to ensure that the departure from the predicted sizes was not due to sequence errors in the construct.

The results of the Reverse Transcriptase-Polymerase Chain Reaction (RT-PCR) confirmed that both the SERA3 and TSERA2 candidate genes were actively transcribed in the blood stage (S3 Fig). By contrast, SSP2/TRAP, a sporozoite stage along with the *Pk*CTRP and *Pk*CSP pre-erythrocytic stage controls, were negative by RT-PCR (S3 Fig).

### SNP analysis: Capturing polymorphic epitopes in target genes

The existence of three major subpopulations of *P*. *knowlesi* have been recently described, two associated with clinical human infections from separate macaque species reservoir hosts and the third from long-term laboratory isolates [50]. The presence of amino acid polymorphisms biased towards a single cluster would likely limit the utility of any reagents generated to function as *P*. *knowlesi*-specific, for all *P*. *knowlesi*-strains. Therefore, we characterised the presence of SNPs associated with the clusters, focussing on non-synonymous positions within the *P*. *knowlesi*-specific truncated constructs. S4 Table summarises both the synonymous and non-synonymous SNPs associated with the three clusters (S5–S8 Tables shows the raw SNP data for all four constructs; SERA3 Ag1, SERA3 Ag2, SSP2/TRAP and TSERA2 respectively). For all antigens, the vast majority of the non-synonymous SNPs lie in regions not covered by the antigen design. By omitting the majority of these cluster-specific SNPs we hoped to avoid segregation of detectable antibodies according to the defined clusters. The relevance of these genetic clusters in the context of immunity, and the potential relevance to host preferences is yet to be defined.

### Serum reactivity to recombinant antigen panel

Serum samples were collected from 97 Malaysian adults and children hospitalised with *P*. *knowlesi* malaria on day of diagnosis (day 0), 7 and 28 days post-treatment. Hospital case samples were assayed by enzyme-linked immunosorbent assay (ELISA) using the *P*. *knowlesi*-specific protein panel. Ethiopian non- *P*. *knowlesi* malaria endemic children’s sera (n = 26) and adult UK malaria naïve sera (n = 29) were used as a *P*. *knowlesi*-negative control panel. The *P*. *knowlesi*-negative malaria endemic controls were all reactive with the *Pv*MSP1-19 antigen due to previous *P*. *vivax* exposure. The malaria naïve controls showed no reactivity to any of the antigens tested (Fig 4, top row and S4 Fig) (SERA3 ag1 OD = 0.124; SERA3 ag2 OD = 0.131; SSP2/TRAP OD = 0.117; TSERA2 ag1 OD = 0.118). With the exception of one weakly positive sample to SERA3 ag 1 and SSP2/TRAP, there was no other detectable antibody reactivity in the control group to the *P*. *knowlesi*-specific antigens (Fig 4). Antibody reactivity to all four antigens appeared to peak at day 7 (Figs 4 and 5 and S4 Fig), although prevalence of antibody responses to SERA3 antigen 1, *Pk*SSP2 and TSERA2 antigen 1 remained relatively low (18.1% (13/72); 33.3% (45/72) and 43.1% (31/72) respectively) (Fig 4, columns 1, 3 and 4), compared to SERA 3 antigen 2 (63.8% (46/72)). The *Pk*SERA3 antigen 2 had a higher prevalence compared to controls at all time-points (p<0.001) (Fig 4 and S4 Fig). Antibody responses measured at day 7 and 28 to SERA3 antigen 2 demonstrated a significant increase when compared to day 0 (p<0.001 for both comparisons), with fold changes as high as 50 observed for some samples (Fig 5). In comparison, the fold changes observed in serum responses to the TSERA2 antigen 1 (day 7 and 28; p = <0.001 and p = 0.005 respectively), SERA3 antigen 1 (day 7; p = 0.008), and *Pk*SSP2 (day 7 and 28; p = 0.001 and p = 0.013), although statistically significant had comparatively lower fold changes with a maximum of 15 (Fig 5).

### Identification of *P*. *knowlesi* exposed individuals

**Fig 6:**
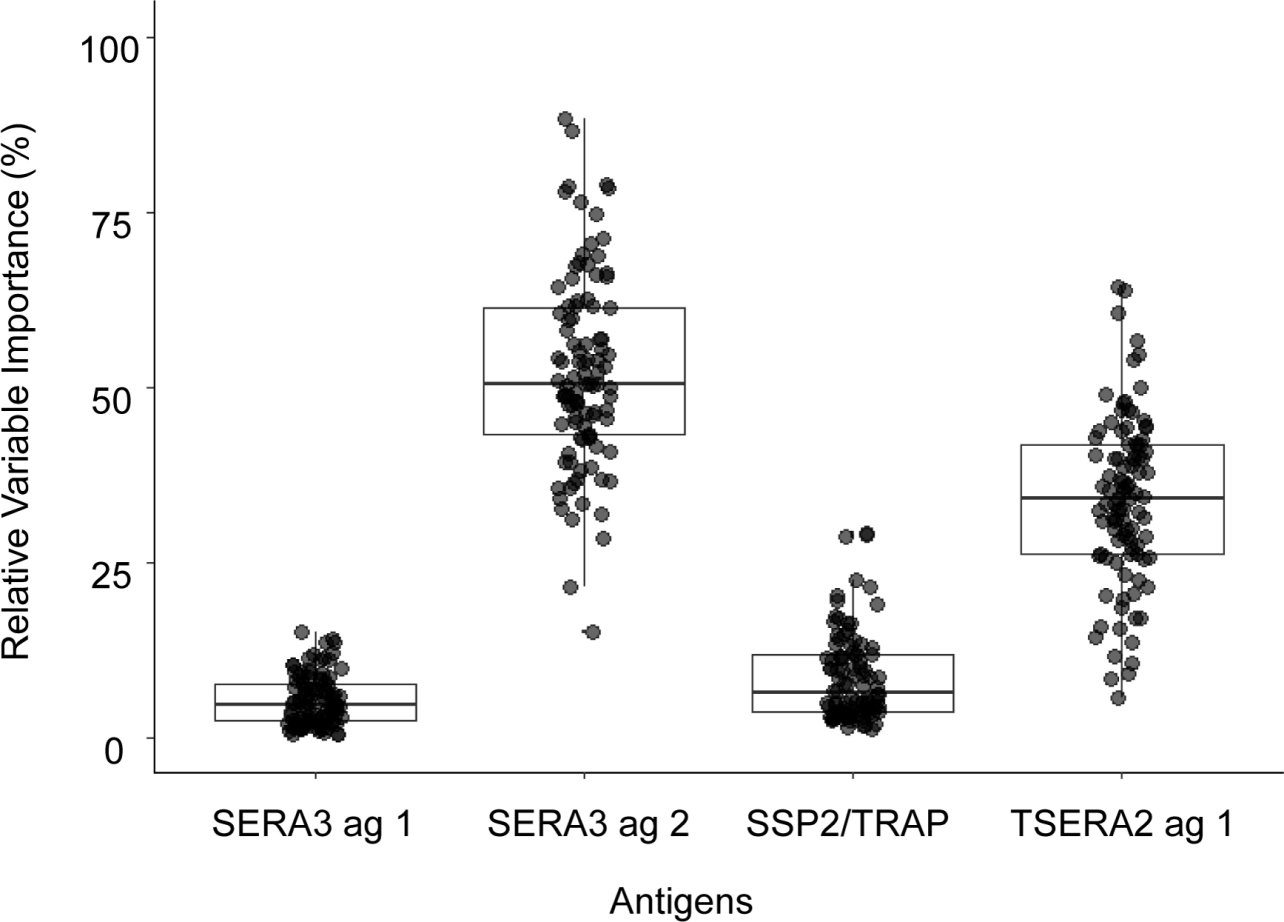
Relative variable importance of responses to each antigen from 100 boosted regression tree models predicting *P*. *knowlesi* seropositivity. Median values for the relative variable importance and interquartile ranges are shown for all antigens tested: SERA3 ag 1 (4.8%; IQR 2.5–7.8%); SERA3 ag 2 (50.4%; IQR 43.3–61.4%); *Pk*SSP2/TRAP (6.5%; IQR 3.7–11.8%) and TSERA ag 1 (34.2%; IQR 26.2–41.8%).

To assess the predictive ability of responses to these antigens to identify *P*. *knowlesi* exposed individuals, we used boosted regression tree analysis, an ensemble modelling method combining aspects of machine learning and statistical analysis shown to have strong predictive performance and reliable identification of variable importance [39]. Similar data-adaptive statistical models are increasingly being used for classification and identification of patterns in large datasets and have previously been applied to identify predictive antigens [51]. Although the samples size is small, boosted regression trees have been used for classification with similarly small training data sets [39]. To further compensate for the small dataset, we fitted 100 models of random samples of equal numbers of sero-positive and sero-negative samples within this training dataset and cross-validated these model predictions. Out of the 100 models fitted for randomly sampled equal numbers of exposed and unexposed individuals, the median classification accuracy was 88.9% (IQR: 86.1–91.3%), calculated as the cross-validated area under the receiver operator curve (AUC). Relative variable importance was calculated for all models. SERA3 antigen 2 contributed most to the models (median relative variable importance: 50.4% (IQR 43.3–61.4%)), followed by TSERA2 antigen 1, *Pk*SSP2/TRAP and SERA3 antigen 1 (Fig 6).

## Discussion

*P*. *knowlesi* is a naturally occurring infection of long-tailed and pig-tailed macaques, historically associated with forested areas of Southeast Asia [52]. Increased deforestation of their natural habitat is thought to have led to increased interaction between macaques and the human population in endemic areas [53]. Changes in village level forest cover and historical forest loss has been associated with an increase in *P*. *knowlesi* clinical cases in Sabah [54], with malaria caused by *P*. *knowlesi* increasingly reported in Southeast Asia [8]. Conversely, there has also been a steady decline in the prevalence of *P*. *falciparum* and *P*. *vivax* infections in the same region [55].

The recent efforts of the malaria community towards achieving malaria elimination means that tools to help monitor the impact and effectiveness of intervention strategies are an urgent requirement [56]. The development of species-specific tools for *P*. *knowlesi* would allow accurate assessment of the levels and geographical limits of infection with this zoonotic species [57]. There is an urgent need to develop a comprehensive discovery strategy to help identify *P*. *knowlesi* unique antigenic markers of exposure in order to further characterise this organism and develop stronger and better identification methods.

Currently, there are no specifically designed biomarkers for the serosurveillance of *P*. *knowlesi* infections. Recombinant proteins are available [*Pk*CSP [58], *Pk*AMA1 [59], *Pk*DBP [60], *Pk*SPATR [15], PkLDH [61], *Pk*1-Cys peroxiredoxin [62], *Pk* knowpains [63], *Pk*MSP1-42 [64], *Pk*MSP1-33 [65], *Pk*MSP1-19 [66], Pk tryptophan-rich antigens (*Pk*Trags) [67], *Pk*MSP3 [68] and *Pk*SBP1 [69], but are limited in number and are generally not species-specific. As a result, their utility as serological diagnostic tools is generally secondary to their original design. The reported level of amino acid sequence conservation to other *Plasmodium* spp. in some currently available *P*. *knowlesi* proteins is > 60% across large stretches of continuous sequence. Such reagents could not be specific to *P*. *knowlesi* [70] and would be unable to reliably discriminate between antibody responses to different parasite species in co-endemic settings.

High levels of amino acid identity (83%) between *Pv*MSP1-19 and *Pk*MSP1-19, meant we were unable to use these reagents to dissect the species-specific immune responses due to the inevitable cross-reactive antibody responses. This is consistent with a proportion (48.9% (45/92)) of the confirmed *P*. *knowlesi*-exposed clinical samples in this study reacting with *Pv*MSP1-19 at day 0, although it is unknown whether these participants had previously been exposed to *P*. *vivax*. However, this limitation simply reflects the paucity of available serological reagents for use in assessing exposure to infection, a deficit this study aims to address. Although the small number of clinical case samples do not give sufficient statistical power to assess either the duration of antibody responses to the panel of antigens or population-level exposure, the *P*. *knowlesi* clinical case samples represent a unique dataset with which to validate the immunogenicity of our antigen panel.

The use of the boosted regression tree model was able to discriminate between *P*. *knowlesi* exposed and unexposed individuals for the purposes of classification of seropositivity rather than to assess individual-level risk factors. While this dataset is sufficient for classification as exposed or unexposed, it is not sufficiently large enough to stratify by age, gender or previously reported malaria status. In order for us to assess these types of risk factors, we would first need to apply an approach (using known negatives, mixture or probability models) to classify antibody responses as sero-positive or sero-negative and then assess risk factors within the population. Based on this result the *Pk*SERA 3 antigen 2 recombinant was used to survey ~2500 samples across three site; Limbuak, Pulau Banggi and Matunggung, Kudat, Sabah, Malaysia and Bacungan, Palawan, the Philippines [71]. One of the key elements from this study using this reagent was the indication of community level patterns of exposure that differed markedly from reported cases, with higher levels of exposure among women and children [71].

The panel of reagents developed for this study focussed on immunologically relevant orthologous targets previously described in *P*. *falciparum*. The serine repeat antigen (SERA) family had previously attracted attention as a source of both drug and vaccine candidates [72]. In *P*. *falciparum*, SERA 5 is the most abundant parasitophorous vacuole protein and is essential to blood stage growth of the parasite [73], with antibodies against this antigen thought to inhibit parasite growth [74]. Although possessing a papain-like enzymatic domain, recent evidence suggests that the protein plays a non-enzymatic role [73]. SERA 3 has also been shown to be a highly immunogenic antigen with an important, although not essential role in the erythrocytic cycle [75] and has also been implicated as having a role in liver stage merozoite release in *P*. *berghei* [76]. Similarly, evidence for the sporozoite surface protein 2 (SSP2/TRAP) suggested an immunogenic antigen involved in protection from disease in mice [77]. Although we were unable to confirm active transcription of SSP2/TRAP due to the lack of available material, we were able to validate active transcription of both the SERA3 and TSERA2 candidate genes. Collectively, the evidence provided by studies on *Plasmodium* supports the design of seroepidemiology tools based on these targets. Despite the targeted approach used in designing the recombinant constructs, the SERA3 antigen 2 construct was by far the most promising candidate. The differences in the performances of the antigens could be due to a number of factors: (1) variation in the inherent immunogenicity of the regions selected, (2) variations in the expression status of the *P*. *knowlesi* antigens compared to *P*. *falciparum* or (3) the loss of immunoreactive epitopes due to the truncation of the protein.

There are a number of potential limitations of the study. The small sample size of the clinical samples used prevented detailed analysis of the samples, such as monitoring the impact of factors such as age, on the profile of reactivity to the reagents under test. In addition, the lack of repeated samples per individual (i.e. longitudinal samples) prevented us from investigating the longevity of antibody responses to each target, across individuals and age groups. The availability of supporting biological information on *P*. *knowlesi*, such as functional data, transcriptional or RNA seq data would have helped with the rational selection of additional candidates for further study and the design recombinant tools.

This is the first study to describe the development a panel of *P*. *knowlesi*-specific serological tools using freely available *in silico* software. We have demonstrated the importance of targeting species-specific reagents at the amino acid level and highlighted the potential of such proteins as serosurveillance tools. Using these tools we have been able to measure specific immune responses to these reagents and described the change in antibody profile following treatment. As such, we have already demonstrated the utility of the SERA3 antigen 2 reagents as a potential seroepidemiological tool. Studies are also currently in development to expand the existing panel of *P*. *knowlesi* species-specific reagents to identify additional serological tools. Beyond this we envisage employing high throughput antigen discovery approaches such as the protein microarray to help identify additional important targets of immunity [51, 78]. Further validation of the SERA3 antigen 2 at the population level has recently been performed [71]. Further studies are also planned to characterise the wider immunoglobulin responses, such as IgG subclasses and IgM, to these and future antigens.

## Supporting information

S1 Checklist PRISMA: Clinical trial in Malaysia, Sabah (P. Knowlesi Trial of Artesunate-mefloquine Versus Chloroquine; www.clinicaltrials.gov: #NCT01708876). (PDF)

S1 Fig **Maximum likelihood phylogenetic analysis of the amino acid sequences of AMA1 (a), MSP1-19 (b), SERA3 (c), TSERA2 (d) and SSP2/TRAP (e) gene sequences between *P*. *knowlesi*, *P*. *falciparum*, *P*. *vivax*, *P*. *malariae* and *P*. *ovale/P*. *simiovale***. Bootstrap values are given in percentages.
(DOCX)

S2 Fig **Amino acid sequences alignments for AMA1 (a), MSP1-19 (b), SERA3 (c), SSP2/TRAP (d) and TSERA2 (e) gene sequences between *P*. *knowlesi*, *P*. *falciparum*, *P*. *vivax*, *P*. *malariae* and *P*. *ovale/P*. *simiovale*. *P*. *knowlesi*-specific sequences selected for development as constructs are highlighted in yellow.** Asterisks indicate fully conserved residues, colons indicates strong residue conservation (>0.5, Gonnet PAM 250 matrix), period indicates weak residue conservation (= <0.5, Gonnet PAM 250 matrix). Conserved cysteine residues are highlighted in green. Blank spaces indicate no residue conservation.
(DOCX)

S3 Fig *Plasmodium knowlesi* candidate gene transcriptional status in parasite mixed blood stage. Panel 1: SERA3; panel 2: SSP2/TRAP; panel 3: TSERA2; panel 4: CTRP; panel 5: CSP. g refers to genomic DNA, RT+ refers to presence of RT enzyme and RT- refers to absence of RT enzyme. Samples were run on a 1.2% agarose gel. The DNA ladder is indicated in bp (Hyperladder 1Kb, Bioline).
(DOCX)

S4 Fig *Plasmodium knowlesi* antigen reactivity to Malaysian hospital case serum samples and negative control serum samples. Dot plot of Malaysian hospital case serum samples from days 0 (n = 92), 7 (n = 72) and 28 (n = 77) of PCR diagnosis and *P*. *knowlesi*-negative control serum samples (Ethiopian *Pv*-positive n = 26; PHE malaria naïve n = 29). Antibody reactivity to the *P*. *knowlesi-*specific antigens (a) SERA3 ag1, (b) SERA3 ag2, (c) SSP2/TRAP and (d) TSERA2 ag1 are shown.
(DOCX)

S1 Table Summary of the percentage amino acid identity between *P*. *knowlesi* and the other *Plasmodium* spp. for all five candidate sequences. (XLSX)

S2 Table *P*. *knowlesi* gene name and ID, primer sequences, primer length, fragment size with and without intron. (XLSX)

S3 Table *P*. *knowlesi* candidate name, primer sequences and primer length. The vector portion of each primer sequence (pGEX-2T) are highlighted in bold and the candidate portion of the sequence in italics. Stop codons are underlined.
(XLSX)

S4 Table Single-nucleotide polymorphism frequencies of Malaysian clinical isolates sequences within *P*. *knowlesi* candidate genes. (XLSX)

S5 Table Synonymous and non-synonymous SNPs associated with the three *P*. *knowlesi* genetic clusters for SERA3 Ag1. (XLSX)

S6 Table Synonymous and non-synonymous SNPs associated with the three *P*. *knowlesi* genetic clusters for SERA3 Ag2. (XLSX)

S7 Table Synonymous and non-synonymous SNPs associated with the three *P*. *knowlesi* genetic clusters for SSP2/TRAP. (XLSX)

S8 Table Synonymous and non-synonymous SNPs associated with the three *P*. *knowlesi* genetic clusters for TSERA2. (XLSX)
