# Supplementary material for: Identification and validation of a novel panel of Plasmodium knowlesi biomarkers of serological exposure
Source: PLoS Negl Trop Dis. 2018 Jun 14;12(6):e0006457. doi: 10.1371/journal.pntd.0006457 (PMC6001954; doi:10.1371/journal.pntd.0006457)
Supplement: S1 Checklist — (PDF) [file pntd.0006457.s001.pdf]

ClinicalTrials.gov Search Results 04/27/2018

	Title	Recruitment	Study Results	Conditions	Interventions	Locations
1	P. Knowlesi Trial of Artesunate-mefloquine Versus Chloroquine	Completed	No Results Available	•Uncomplicated Plasmodium Knowlesi Malaria	•Drug: Artesunate-mefloquine<br>•Drug: Chloroquine<br>•Drug: Primaquine	•Kota Marudu District Hospital, Kota Marudu, Sabah, Malaysia<br>•Kudat District Hospital, Kudat, Sabah, Malaysia<br>•Pitas District Hospital, Pitas, Sabah, Malaysia

U.S. National Library of Medicine | U.S. National Institutes of Health | U.S. Department of Health & Human Services
