# Supplementary material for: Identification and validation of a novel panel of Plasmodium knowlesi biomarkers of serological exposure
Source: PLoS Negl Trop Dis. 2018 Jun 14;12(6):e0006457. doi: 10.1371/journal.pntd.0006457 (PMC6001954; doi:10.1371/journal.pntd.0006457)

**Supporting Information**

**Supplementary Figure 1:** Maximum likelihood phylogenetic analysis of the amino acid sequences of AMA1 (a), MSP1-19 (b), SERA3 (c), TSERA2 (d) and TRAP/SSP2 (e) gene sequences between *P. knowlesi, P. falciparum, P. vivax, P. malariae* and *P. ovale/P. simiovale*. Bootstrap values are given in percentages.


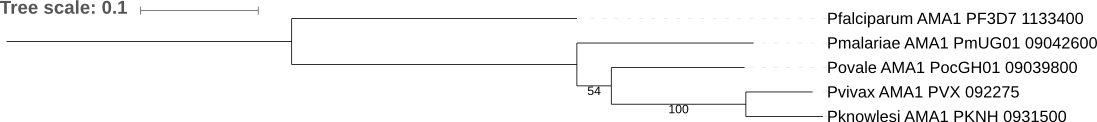
Supplementary Figure 1a

Supplementary Figure 1b


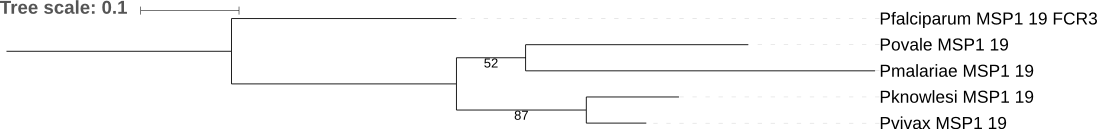


Supplementary Figure 1c


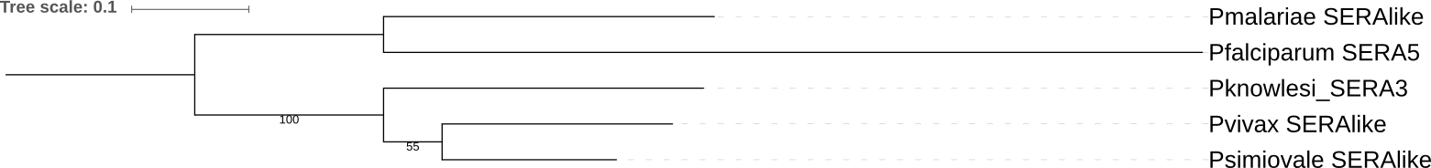


Supplementary Figure 1d


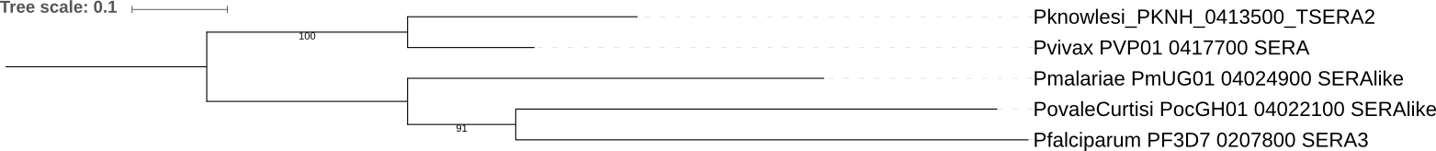


Supplementary Figure 1e
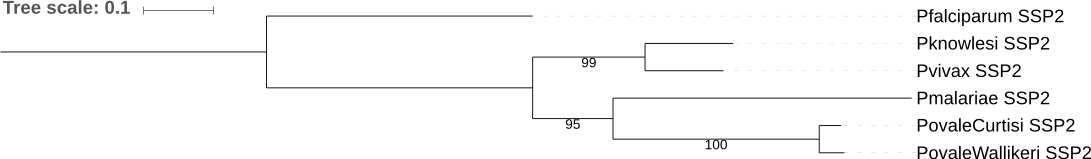

Supplement: S1 Fig — Maximum likelihood phylogenetic analysis of the amino acid sequences of AMA1 (a), MSP1-19 (b), SERA3 (c), TSERA2 (d) and SSP2/TRAP (e) gene sequences between P. knowlesi, P. falciparum, P. vivax, P. malariae and P. ovale/P. simiovale. Bootstrap values are given in percentages. (DOCX) [file pntd.0006457.s002.docx]
