# Supplementary material for: Identification and validation of a novel panel of Plasmodium knowlesi biomarkers of serological exposure
Source: PLoS Negl Trop Dis. 2018 Jun 14;12(6):e0006457. doi: 10.1371/journal.pntd.0006457 (PMC6001954; doi:10.1371/journal.pntd.0006457)
Supplement: S2 Fig — Amino acid sequences alignments for AMA1 (a), MSP1-19 (b), SERA3 (c), SSP2/TRAP (d) and TSERA2 (e) gene sequences between P. knowlesi, P. falciparum, P. vivax, P. malariae and P. ovale/P. simiovale. P. knowlesi-specific sequences selected for development as constructs are highlighted in yellow. Asterisks indicate fully conserved residues, colons indicates strong residue conservation (>0.5, Gonnet PAM 250 matrix), period indicates weak residue conservation (= <0.5, Gonnet PAM 250 matrix). Conserved cysteine residues are highlighted in green. Blank spaces indicate no residue conservation. (DOCX) [file pntd.0006457.s003.docx]

**Supporting Information**

**Supplementary Figure 2:** Amino acid sequences alignments for AMA1 (a), MSP1-19 (b), SERA3 (c), TRAP/SSP2 (d) and TSERA2 (e) gene sequences between *P. knowlesi, P. falciparum, P. vivax, P. malariae* and *P. ovale/P. simiovale*. *P. knowlesi*-specific sequences selected for development as constructs are highlighted in yellow. Asterisks indicate fully conserved residues, colons indicates strong residue conservation (>0.5, Gonnet PAM 250 matrix), period indicates weak residue conservation (=<0.5, Gonnet PAM 250 matrix). Conserved cysteine residues are highlighted in green. Blank spaces indicate no residue conservation.

**Supp. Fig 2a: Apical Membrane Antigen 1 (AMA1)**

Pfalciparum_AMA1_PF3D7_1133400 MRKLYCVLLLSAFEFTYMINFGRGQNYWEHPYQNSDVYRPINEHREHPKEYEYPLHQEHT

Pmalariae_AMA1_PmUG01_09042600 MKKLYYILLLST---QYLIHVYA-------------------------------------

Povale_AMA1_PocGH01_09039800 MKKIYYIFLLSA---HYLINVGK-------------------------------------

Pknowlesi_AMA1_PKNH_0931500 MNKIYYILFLSA---QCLVHMGK-------------------------------------

Pvivax_AMA1_PVX_092275 MNKIYYIIFLSA---QCLVHIGK-------------------------------------

*.*:* :::**: :::.

Pfalciparum_AMA1_PF3D7_1133400 YQQEDSGEDENTLQHAYPIDHEGAEPAPQEQNLFSSIEIVERSNYMGNPWTEYMAKYDIE

Pmalariae_AMA1_PmUG01_09042600 -----SPRNAKHGRLNGSG-GTLIEKGQ----------VVERSTRMSNPWKKYMEKYDVE

Povale_AMA1_PocGH01_09039800 -----CTRNQKQGRLTRSG-SAMVEKNP----------TIERSTRMINPWKKYMEKFDVE

Pknowlesi_AMA1_PKNH_0931500 -----CERNQKTTRLTRSANNASLEKGP----------IIERSIRMSNPWKAFMEKYDLE

Pvivax_AMA1_PVX_092275 -----CGRNQKPSRLTRSANNVLLEKGP----------TVERSTRMSNPWKAFMEKYDIE

. : : . . * :*** * ***. :* *:*:*

Pfalciparum_AMA1_PF3D7_1133400 EVHGSGIRVDLGEDAEVAGTQYRLPSGKCPVFGKGIIIENSNTTFLTPVATGNQYLKDGG

Pmalariae_AMA1_PmUG01_09042600 KTHGAGVRVDLGEDAEVKNSKYRIPGGKCPVFGKGIIIENSNVDFLTPVATGNRNLKSGG

Povale_AMA1_PocGH01_09039800 KTHGSGIRVDLGEDAEVKNSKYRIPSGRCPVFGKGITIENSEVSFLKPVATGNEKLKSGG

Pknowlesi_AMA1_PKNH_0931500 RAHNSGIRIDLGEDAEVGNSKYRIPAGKCPVFGKGIVIENSNVSFLTPVATGAQRLKEGG

Pvivax_AMA1_PVX_092275 RTHSSGVRVDLGEDAEVENAKYRIPAGRCPVFGKGIVIENSDVSFLRPVATGDQKLKDGG

.*.:*:*:******** .::**:*.*.******** ****:. ** ***** **.**

Pfalciparum_AMA1_PF3D7_1133400 FAFPPTEPLMSPMTLDEMRHFYKDNKYVKNLDELTLCSRHAGNMIPDNDKNSNYKYPAVY

Pmalariae_AMA1_PmUG01_09042600 FAFPATDDHISPVTIEVLRKRYEEHADLMNLNDLSLCSKHASSFVISDDLNTSYRHPAVY

Povale_AMA1_PocGH01_09039800 FAFPLTDYHISPISLQNLKRRYNENVELMKLNDMSLCAKHASSFVISEDQNTTYRHPAVY

Pknowlesi_AMA1_PKNH_0931500 FAFPNADDHISPITIANLKERYKENADLMKLNDIALCKTHAASFVIAEDQNTSYRHPAVY

Pvivax_AMA1_PVX_092275 FAFPNANDHISPMTLANLKERYKDNVEMMKLNDIALCRTHAASFVMAGDQNSSYRHPAVY

**** :: :**::: :. *::: : :*::::** **..:: * *:.*.:****

Pfalciparum_AMA1_PF3D7_1133400 DDKDKKCHILYIAAQENNGPRYCNKDESKRNSMFCFRPAKDISFQNYTYLSKNVVDNWEK

Pmalariae_AMA1_PmUG01_09042600 DEKTKTCYILYLSAQENIGPRYCSKDAADKDTMFCFKPAKTDNFKHYAYLSKNVVSDWDV

Povale_AMA1_PocGH01_09039800 DEKEQTCYILYLSAQENLGPRYCSNDAADKDSIFCFKPEKNESFQNYVYLSKNLRDDWSS

Pknowlesi_AMA1_PKNH_0931500 DEKNKTCYMLYLSAQENMGPRYCSPDSQNKDAMFCFKPDKNEKFDNLVYLSKNVSNDWEN

Pvivax_AMA1_PVX_092275 DEKEKTCHMLYLSAQENMGPRYCSPDAQNRDAVFCFKPDKNESFENLVYLSKNVRNDWDK

*:* :.*::**::**** *****. * ..:::***.* * .*.: .*****: .:*.

Pfalciparum_AMA1_PF3D7_1133400 VCPRKNLQNAKFGLWVDGNCEDIPHVNEFPAIDLFECNKLVFELSASDQPKQYEQHLTDY

Pmalariae_AMA1_PmUG01_09042600 KCPRKSLGVAKFGLWVDGNCEEIPSVKAFYADNLTECNRIVFEASASDQPTQYEENMTDY

Povale_AMA1_PocGH01_09039800 KCPRNNLTNSKFGLWVDGNCEDIPYVKEFQANTLRECNRIVFEASASDQPRQYEEELTDY

Pknowlesi_AMA1_PKNH_0931500 KCPRKNLGNAKFGLWVDGNCEEIPYVNEVEARSLRECNRIVFEASASDQPRQYEEELTDY

Pvivax_AMA1_PVX_092275 KCPRKNLGNAKFGLWVDGNCEEIPYVKEVEAEDLRECNRIVFGASASDQPTQYEEEMTDY

***:.* :***********:** *: . * * ***.:** ****** ***: :***

Pfalciparum_AMA1_PF3D7_1133400 EKIKEGFKNKNASMIKSAFLPTGAFKADRYKSHGKGYNWGNYNTETQKCEIFNVKPTCLI

Pmalariae_AMA1_PmUG01_09042600 KKLEQGFRDNNPDMIKGAFLPVGAFNANFNKSKGKGFNWGNYDKINKKCFIFNVKPTCLI

Povale_AMA1_PocGH01_09039800 EKIQEGFRQNNPDMIKGAFFPVGAYKSDNFKSRGKGFNWGNFDIVNKKCYIFSAKPTCLI

Pknowlesi_AMA1_PKNH_0931500 EKIQEGFRQNNRDMIKSAFLPVGAFNSDNFKSKGRGYNWANFDSVNNKCYIFNTKPTCLI

Pvivax_AMA1_PVX_092275 QKIQQGFRQNNREMIKSAFLPVGAFNSDNFKSKGRGFNWANFDSVKKKCYIFNTKPTCLI

:*:::**.::* .***.**:*.**:::: **.*.*:**.*:: .:** **..******

Pfalciparum_AMA1_PF3D7_1133400 NNSSYIATTALSHPIEVENNFPCSLYKDEIMKEIERESKRIKLNDNDDEGNKKIIAPRIF

Pmalariae_AMA1_PmUG01_09042600 NNKDFIATTALSHPEEVQEDFPCDIYKNEIEKELKRNSGNVKLYSLDGE---KIVLPRIF

Povale_AMA1_PocGH01_09039800 NDKNYIATTALSHPEDVERNFPCEIYKNEIEKEIEKQNRKAKLYSTDGD---RVVLPRIF

Pknowlesi_AMA1_PKNH_0931500 NDKNFFATTALSHPQEVDNEFPCSIYKDEIEREIKKQSRNMNLYSVDKE---RIVLPRIF

Pvivax_AMA1_PVX_092275 NDKNFIATTALSHPQEVDLEFPCSIYKDEIEREIKKQSRNMNLYSVDGE---RIVLPRIF

*:..::******** :*: :***.:**:** .*::.:. . :* . * : .:: ****

Pfalciparum_AMA1_PF3D7_1133400 ISDDKDSLKCPCDPEMVSNSTCRFFVCKCVERRAEVTSNNEVVVKEEYKDEYADIPEHKP

Pmalariae_AMA1_PmUG01_09042600 ISNNKDSLNCPCEPEKITNSSCDFYLCNCVEKRAEIKENNEVVIKDEFKEEYEYNEGNS-

Povale_AMA1_PocGH01_09039800 ISDDKDSLKCPCEPERITNSTCNYYVCNCVEKRAEIKENNEVIIKDEFKEDYENEEGENT

Pknowlesi_AMA1_PKNH_0931500 ISTDKESIKCPCEPEHISNSTCNFYVCNCVEKRAEIKENNEVIIKEEFKEDYENPDGKH-

Pvivax_AMA1_PVX_092275 ISNDKESIKCPCEPERISNSTCNFYVCNCVEKRAEIKENNQVVIKEEFRDYYENGEEKS-

** :*:*::***:** ::**:* :::*:***.***:..**:*::*:*:.: *

Pfalciparum_AMA1_PF3D7_1133400 TYDKMKIIIASSAAVAVLATILMVYLYKRKGNAEKYDKMDEPQDYGKS-NSRNDEMLDPE

Pmalariae_AMA1_PmUG01_09042600 NNKKTLIIIGLAGGVGILALASSFFFFKKKTENEKYDKMDQADVYGKS-TTRKDEMLDPE

Povale_AMA1_PocGH01_09039800 NRQRTIIIIGLAGGVAVLGCASFFFFFKKKAQGKEYDKMDQTDGYGKP-KSRKDEMLDPE

Pknowlesi_AMA1_PKNH_0931500 KKKMLLIIIGVTGAVCVVAVASLFY-FRKKAQDDKYDKMDQAEAYGKTANTRKDEMLDPE

Pvivax_AMA1_PVX_092275 NKQMLLIIIGITGGVCVVALASMAY-FRKKANNDKYDKMDQAEGYGKP-TTRKDEMLDPE

. . ***. :..* ::. : :..* : .:*****:.: ***. .:*:*******

Pfalciparum_AMA1_PF3D7_1133400 ASFWGEEKRASHTTPVLMEKPYY

Pmalariae_AMA1_PmUG01_09042600 ASFWGEEKRASHTTPVLMEKPYY

Povale_AMA1_PocGH01_09039800 ASFWGEEKRASHTTPVLMEKPYY

Pknowlesi_AMA1_PKNH_0931500 ASFWGEDKRASHTTPVLMEKPYY

Pvivax_AMA1_PVX_092275 ASFWGEDKRASHTTPVLMEKPYY

******:****************

**Supp. Fig 2b: Merozoite Surface Protein (MSP) 1-19**

Pfalciparum_MSP1_19_FCR3 -NISQHQCVKKQCPQNSGCFRHLDEREECKCLLNYKQEGDKCVENPNPTCNENNGGCDAD

Pmalariae_MSP1_19 NISAKHACTETKYPENAGCYRYEDGKEVWRCLLNYKLVDGGCVEDEEPSCQVNNGGCAPE

Povale_MSP1_19 -MGSKHKCIDITYPDNAGCYRFSDGREEWRCLLNFKKVGETCVPNNNPTCAENNGGCDPT

Pknowlesi_MSP1_19 NMSSAHKCIDTNVPENAACYRYLDGTEEWRCLLGFKEVGGKCVPAS-ITCEENNGGCAPE

Pvivax_MSP1_19 -MSSEHTCIDTNVPDNAACYRYLDGTEEWRCLLTFKEEGGKCVPASNVTCKDNNGGCAPE

: * * . *:*:.*:*. * * .*** :* . ** :* ***** .

Pfalciparum_MSP1_19_FCR3 AKCTEEDSGSNGKKITCECTKPDSYPLFDGIFCSSSN

Pmalariae_MSP1_19 ANCTKGDD----NKIVCACNAPYSEPIFEGVFCGSSS

Povale_MSP1_19 ADCAESEN----NKITCTCTGQ-NESFFEGVFCGSSS

Pknowlesi_MSP1_19 AECTMDDK----KEVECKCTKEGSEPLFEGVFCSSSS

Pvivax_MSP1_19 AECKMTDS----NKIVCKCTKEGSEPLFEGVFCSSSS

*.* :. ::: * *. . .:*:*:**.**.

**Supp. Fig 2c: Serine Repeat Antigen (SERA) 3 protein**

**Antigen 1**

Pfalciparum_SERA5 MKSYISLFFILCVIFNKNVIKCT--GESQTGNTG---------GGQAGNTGGDQAGSTGG

Pmalariae_SERAlike MKYGILYIFMICISFGSNTIKCTTVSVSDNRGNEASEQPLQPAQPGPQTHEPSNSQVQNS

Pknowlesi_SERA3 MKSSFLLLLALCATYGNNLAICTTEGTAQSGVSSDSQHSLSSSETETGSHGAPGAEAQSV

Pvivax_SERAlike MKSSVLLLLALGATYGNNVAMCT--ATPPSGGPHASLPNPGGPGTGAENQGQSQAGQQLP

Psimiovale_SERAlike MKSSFLLLLALGTAYGNNVVICTTGQTPSSGESGVASSSSSGPGTGSDNQQESGQGPQPE

** . :: : :..* ** . . . .

Pfalciparum_SERA5 SPQGSTG---------------ASPQGSTGASPQGSTGASQPGSSEPS---NPVSSGHSV

Pmalariae_SERAlike SNPNISDLTVTTPPVAQNLSHETPKNGSSQSPPQNGPLLSSPSAVNNGQ--PNVSSGGAV

Pknowlesi_SERA3 SPEGGQD---AVHSTN-ESAESDAESPTEPNPPQEDGTSNEDGNGGHSESSAPSVPGGSA

Pvivax_SERAlike SPSGEPG---AASPTHTPSPGPLPPNPAQPNSLPAVETLSQQGGGAPAASSNALTAGGNV

Psimiovale_SERAlike SAGNGQN---VEESTNHQQPVPGAPNSAGSNLSQGGATIKSNGDGNPSAISPAPNAEGSV

* . . . . : .. . . . .

Pfalciparum_SERA5 STVSVSQTSTSSEK-------------------QDTIQVKSALLKDYMGLKVTGPCNENF

Pmalariae_SERAlike SP-NLSSAGNSNGATQLSAESQNGAVSPKVPNYHNMAKIESALLKNHTGVRITGPCNEEV

Pknowlesi_SERA3 SPSSVENSNEQAAGTQLQVA-------------PQKAQVKSALLKNFTGVKVTGPCDTEV

Pvivax_SERAlike PPGSQVNSGEQGGATQLQAT-------------PKKAELQSSLLKNFTGVKVTGPCDTEV

Psimiovale_SERAlike SPSSSGSTRGPGGATQLQAS-------------HKKAELQSALLKNFTGVKVTGPCDTEV

.. . .: . :::*:***:. *:.:****: :.

**Antigen 2**

Pfalciparum_SERA5 IYDYYLKASPEFYHNLYFKNFNVGKKNLFSEKEDNENN---KKLGNNYIIFGQDT-----

Pmalariae_SERAlike LYNYYLKTSPDFYSNLYFNSLSAEKANDLST----------NKVLDQMTVHGQAVEESSE

Pknowlesi_SERA3 LHSYYLKNSPDFYKNLYYNALDGECGNAPCNTVEGQDA-PGEKATDQVGASGAGVATVTT

Pvivax_SERAlike LYSYHLKTSPDFYKNLYYNAVGGEKGSVLSNAVQGQDTPPGEEALPGAKVDGGGT-----

Psimiovale_SERAlike LYSYHLNSSPDFYKNLYYNALGEKSGSALSHAVHGQDA-PQEEGESLGTVVGEGISESTV

::.*:*: **:** ***:: .. . . :: *

Pfalciparum_SERA5 ----------------------------------------------------AGSGQSGK

Pmalariae_SERAlike ----------------------------------------------------GTSGQHGQ

Pknowlesi_SERA3 ----------------------------------------------TGTGAAPGTGAEAK

Pvivax_SERAlike ------------------------------------------------------PGPTGP

Psimiovale_SERAlike QEVQQTQLQVPSVMSTNPRVEGQQEQAVVVDQASSHQRAEQVEASTLGAANTQESGPKVT

.*

Pfalciparum_SERA5 ESNT--------------------------------------------------------

Pmalariae_SERAlike HGQP--------------------------------------------------------

Pknowlesi_SERA3 AGAEAGAGAETE--ATKATEVLEPKEQQAQSQVTVVENSVSKDQPQPQPQPQPQPQPQLQ

Pvivax_SERAlike EGQPQPHASSVG--------GQIP----QESRQEVEEKQVVDGQ----------------

Psimiovale_SERAlike EGAQNGDAQSTGIAGVQTAPGTAPAIGAQTAQNTVGGETAAGGQGEGSVVSQLAADGTDS

.

Pfalciparum_SERA5 ---------------------------------------------------------ALE

Pmalariae_SERAlike -------------------GQPESTS-----------------SSETVAESSA---QGLD

Pknowlesi_SERA3 QEQQQEQQQEQQQEQQQEQQQQQEQQQQQPQPPQPALQDLTNEHSPHIGETEVKVEPEGE

Pvivax_SERAlike ---------PQPVTVE---GHPPSQPLQPTSPLQQSTQPGVGGAASTPLSQGT---PGQA

Psimiovale_SERAlike RGITTDISSPQAQTLQPPGGQGVSTPAQPPSP-QQPTEP---VHASTSLVTGT---PGAA

Pfalciparum_SERA5 SAGTSNEVSERVHVYHILKHIKDGKIRMGMRKYIDTQDVNKKHSCTRSYAFNPENYEKCV

Pmalariae_SERAlike SAASDVPDVQKFEVVHILKHIKNSKSKTTLVKYDYYYDFG-DHACSRTQASNPEKLGDCI

Pknowlesi_SERA3 NSNAELQKAKMVQIIHVLKHIKQTKMVTRVVTYQGNYELG-EHSCSRTEASSVEKLDECI

Pvivax_SERAlike NSNGEAENANISQIIHVLKHIKKTKMVTRIVTYEGEYDLG-DHSCSRTQASSLEKLDDCI

Psimiovale_SERAlike NPNAGVQSAKISQIIHVLKHIKQTKMVTRVVTYEGEYELG-DQSCSRTQALSLEKLDECI

.. : : *:*****. * : .* :.. .::*:*: * . *: .*:

**Supp. Fig 2d: Sporozoite Surface Protein (SSP) 2**

Pfalciparum_SSP2 ENPPNPD--IPEQEPNIPEDSEKEVPSD------VPKNPEDD---RE-------------

Pknowlesi_SSP2 TVPDESN--VIPVPPTVPGGSNSEFSSDVENAAQYPENPENPEN-PENSENPENPENQ--

Pvivax_SSP2 SVPDESN--VLPLPPAVPGGSSEEFPAD------VQNNPD--------------------

Pmalariae_SSP2 PNSESKDKLLPSDKEDEENKNNSNLPEG------LEERPQEGESLPVEPEGNENVEDNFP

PovaleWallikeri_SSP2 FVPDEEV--LPEAPANVPEERGNDVPEE------FPQNPENEQNVPE-------------

PovaleCurtisi_SSP2 FILDEEN--LPEAPANVPEG-GNDVPEE------FPQNPENEQNVPE-------------

. : .:.. :.*:

Pfalciparum_SSP2 -ENFDIPKKPENKH-------DN--QNNLPNDKSDR---SIPYSPLPPKVLDNERKQSDP

Pknowlesi_SSP2 NNPEDFPMEPDMSA-------DN--KINEPTNPSDSGQ-GIPENVIPTPINNEKDIINKN

Pvivax_SSP2 -SPEELPMEQEVPQ-------DN--NVNEPERSDSNGY-GVNEKVIPNPLDNERDMANKN

Pmalariae_SSP2 QAPNDLPGKQGQPDILNPDGGPNFGNNEHPGSPSNNDYSGKAYTHIPSPIGNEKNRSNYN

PovaleWallikeri_SSP2 -KPNDLPIEQEKPQDDGNNKVDY--KKNDMYKPEKGGYVIENDHRAPKPSNSYSDSKGKA

PovaleCurtisi_SSP2 -KPNDLPIEQEKPQDDGNNKGDH--KKSDIHVPETAGYVIENGHRVPKPLVNYSDDKGKA

::* : : . . * . .

Pfalciparum_SSP2 QSQDNNGNRHVPNSEDRETRPHGRNNENRSYNRKYNDTP---KHPEREEHEKPDNNKKKG

Pknowlesi_SSP2 KAVYPNGS---NQSHDRYPKPHRNAGGYDNNPNANSDIP---EGPFSSEEEQPEDKGKK-

Pvivax_SSP2 KTVHPDRK---DSARDRYARPHGSTHVNNNRANENSDIP---NNPVPSDYEQPEDKAKK-

Pmalariae_SSP2 H--NYSKSPNNNGPEDRVARPHKVDTNTESPRDSYNANPEYDETRESPNYEQREDNGKR-

PovaleWallikeri_SSP2 QSMNYSSNQYNNIPEERYPKPHKSIGRKDNSRNNYPSAP---YTPEEPTDDEYANKGKK-

PovaleCurtisi_SSP2 QSMNNEGNKNKNTSEERYPKPHKSIGRNDNSRKNYPSAP---YTPEEPTDDEYANKGKK-

: . . . :* ..** . * :: :: *.

**Supp. Fig 2e: Truncated Serine Repeat Antigen (TSERA) 2 protein**

Pknowlesi_PKNH_0413500_TSERA2 MKARLSLILILCAVCRECTVRCTDTAIN-------QGQDAVQQPEESLSQDASDNSLPGQ

Pvivax_PVP01_0417700_SERA MKARLSLILILCVVCRDCAVRCTGTTEA------QGAVEGAKGPKPEAEEAGAGKEEGAN

PovaleCurtisi_PocGH01_04022100_S MKSHVSIIVTLYGIFSIHVTQCTGGGAHWPPHNMHTSNPGNGNKTGDSAIPPSSGNLST-

Pfalciparum_PF3D7_0207800_SERA3 MKFSISLFLILCVLFCKNDIKCTTVDES--------TKEGSQNPKNSSSTTPASGSQKG-

Pmalariae_PmUG01_04024900_SERAli MRSCISLLLTIA---------CASDSNN--------GAPGSTG--NSSTTISSSNSERS-

*. :*::: : *: . . :. .

Pknowlesi_PKNH_0413500_ TSERA2 PVASPNGADQA----EVSQLQEGAVESSSNSADASNPNANADVTDVEGEKAATPSEGS--

Pvivax_PVP01_0417700_SERA VGEAGTGGPGADGGTEAGARAEEGEGAGTEAEPARGPEPEAGGEGINRDAAGNQREGQLE

PovaleCurtisi_PocGH01_04022100_S --SA-SGSTGS----ILPECTGESKGEDVKGGACSNSAPIEDLTLDRIEVVSIDKDGE--

Pfalciparum_PF3D7_0207800_SERA3 --SS-SESPGS----SVEKQSQESNKESTNGGNVVSQGTPANTFGQNSNNPSDSPQGT--

Pmalariae_PmUG01_04024900_SERAli --ASPSESPSS------------GGQATSNGG--------SNLVGSSTAVPSNPQPSQ--

: . . : . :. . . .

Pknowlesi_PKNH_0413500_ TSERA2 -------------------------------------------------------KEGMQ

Pvivax_PVP01_0417700_SERA APSDSARPGAIPQVAPRDTVETSSDAADSSSPDQNPLPGADNTKVGNAATPPEGAKEETQ

PovaleCurtisi_PocGH01_04022100_S -----------------DVEEGQNNEEKTENYIQKIIHSRTHSTVSHIRERTTDEPNIIE

Pfalciparum_PF3D7_0207800_SERA3 -----------------ST--------------------------------LPSPPKSID

Pmalariae_PmUG01_04024900_SERAli -----------------DV--------------------------------STSIANKAK

: .

Pknowlesi_PKNH_0413500_ TSERA2 VKSSLLKGYKGVKVTGPCSASFLVFFAPYLFIDVDADSSNIYLGTDLNDLE---------

Pvivax_PVP01_0417700_SERA VKSSLLKGHKGVKVTGPCGASFLVFFAPYLFIDVDTDSSNVYLGTDLSDLE---------

PovaleCurtisi_PocGH01_04022100_S IKSALLRDYNGVKVTGPCKAVFQMFLVPHITVNVETNKNSITLGPKLVQAHKKERVTTDG

Pfalciparum_PF3D7_0207800_SERA3 VKSAFLKHYKGVKVTGSCNANFQLFLVPHIFINVETKENNIQLDVKFLK-----------

Pmalariae_PmUG01_04024900_SERAli IQSALLTDSNGVMVTGPCNEIFQVFFVPNIFINVQTDKNTVEMGNKFKS-----------

::*::* :** ***.* * :*:.* : ::*::....: :. .: .

Pknowlesi_PKNH_0413500_ TSERA2 -------ITEKMGKGKDE---KNKCQEGKT--FKFVAFVVNDHLTIKWKVYDSEDQTPTP

Pvivax_PVP01_0417700_SERA -------VTEKMGIQDNG---KNKCEDKKT--FKFVALIGEDHLTIKWKVYDPSVKTPTP

PovaleCurtisi_PocGH01_04022100_S VDAYTEHIEKGLMFEKEEKKLLNKCADGKS--FKFVLFIEGNKLTVKWKVYD-ATEAENS

Pfalciparum_PF3D7_0207800_SERA3 -------LTKRIDFAKDKSMLKNKCESGKNQTFKFVLYFKDDILTIKWKVYEEKSATPQK

Pmalariae_PmUG01_04024900_SERAli -------LSNSITLKTFEYS-KNECAGGKT--FKFVALIQENKLTLKWKVYDAPNQNKTT

: : : *:* *. **** . : **:*****:
