# Supplementary material for: Identification and validation of a novel panel of Plasmodium knowlesi biomarkers of serological exposure
Source: PLoS Negl Trop Dis. 2018 Jun 14;12(6):e0006457. doi: 10.1371/journal.pntd.0006457 (PMC6001954; doi:10.1371/journal.pntd.0006457)
Supplement: S3 Fig — Panel 1: SERA3; panel 2: SSP2/TRAP; panel 3: TSERA2; panel 4: CTRP; panel 5: CSP. g refers to genomic DNA, RT+ refers to presence of RT enzyme and RT- refers to absence of RT enzyme. Samples were run on a 1.2% agarose gel. The DNA ladder is indicated in bp (Hyperladder 1Kb, Bioline). (DOCX) [file pntd.0006457.s004.docx]

**Supporting Information**

**Supplementary Figure 3: *Plasmodium knowlesi* candidate gene transcriptional status in parasite mixed blood stage.** Panel 1: SERA3; panel 2: SSP2; panel 3: TSERA2; panel 4: CTRP; panel 5: CSP. g refers to genomic DNA, RT+ refers to presence of RT enzyme and RT- refers to absence of RT enzyme. Samples were run on a 1.2% agarose gel. The DNA ladder is indicated in bp (Hyperladder 1Kb, Bioline).

**
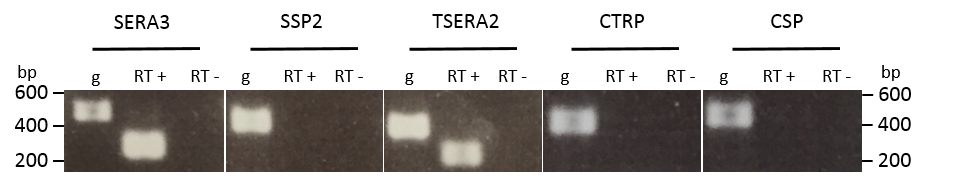
**
