# Supplementary material for: Identification and validation of a novel panel of Plasmodium knowlesi biomarkers of serological exposure
Source: PLoS Negl Trop Dis. 2018 Jun 14;12(6):e0006457. doi: 10.1371/journal.pntd.0006457 (PMC6001954; doi:10.1371/journal.pntd.0006457)
Supplement: S4 Fig — Dot plot of Malaysian hospital case serum samples from days 0 (n = 92), 7 (n = 72) and 28 (n = 77) of PCR diagnosis and P. knowlesi-negative control serum samples (Ethiopian Pv-positive n = 26; PHE malaria naïve n = 29). Antibody reactivity to the P. knowlesi-specific antigens (a) SERA3 ag1, (b) SERA3 ag2, (c) SSP2/TRAP and (d) TSERA2 ag1 are shown. (DOCX) [file pntd.0006457.s005.docx]

**Supporting Information**

**Supplementary Figure 4: *Plasmodium knowlesi* antigen reactivity to Malaysian hospital case serum samples and negative control serum samples.** Dot plot of Malaysian hospital case serum samples from days 0 (n=92), 7 (n=72) and 28 (n=77) of PCR diagnosis and *Pk*-negative control serum samples (Ethiopian *Pv*-positive n=26; PHE malaria naïve n=29). Antibody reactivity to the *P. knowlesi-*specific antigens (a) SERA3 ag1, (b) SERA3 ag2, (c) SSP2 and (d) TSERA2 ag1 are shown.

Supplementary Figure 4a
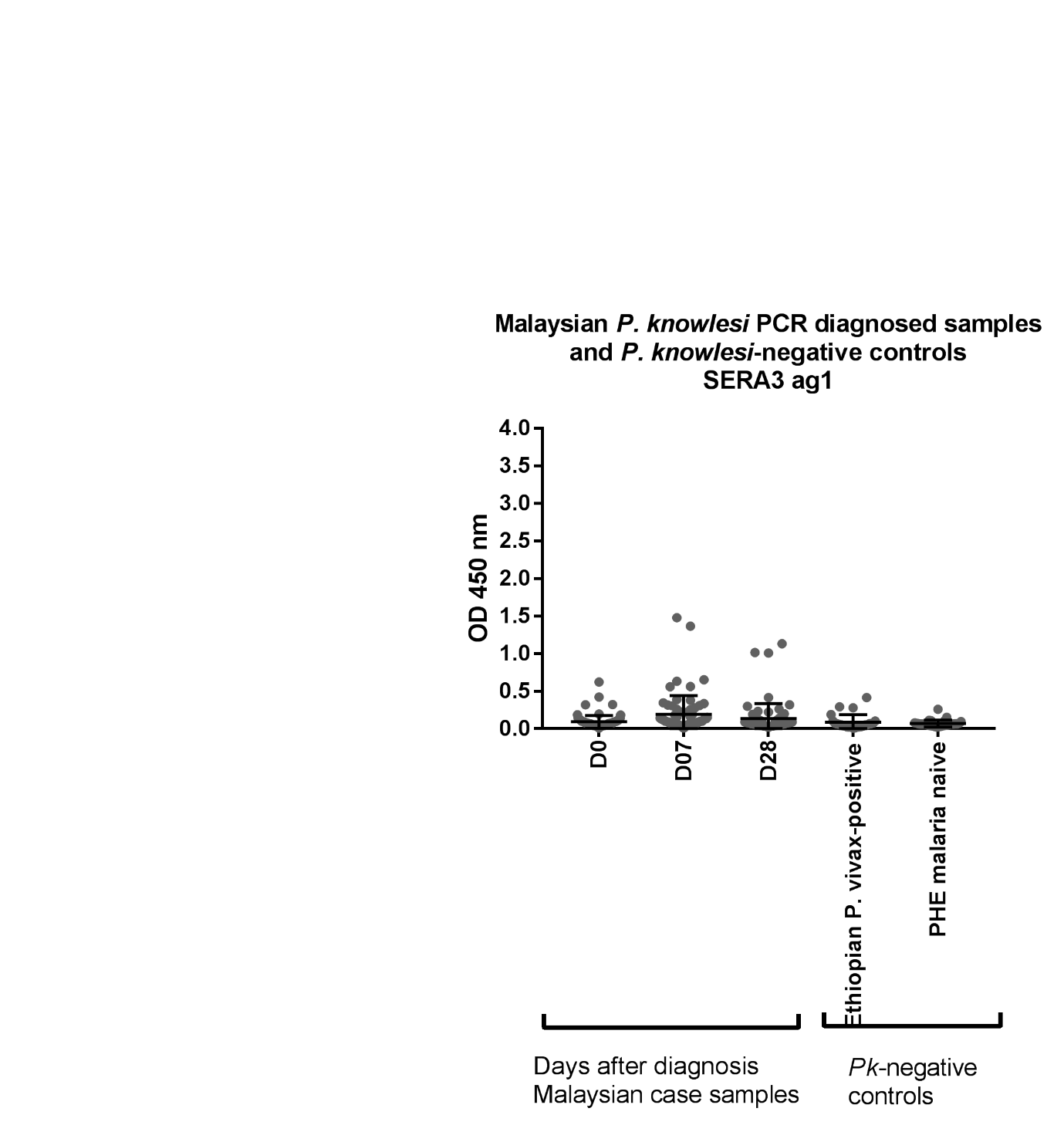


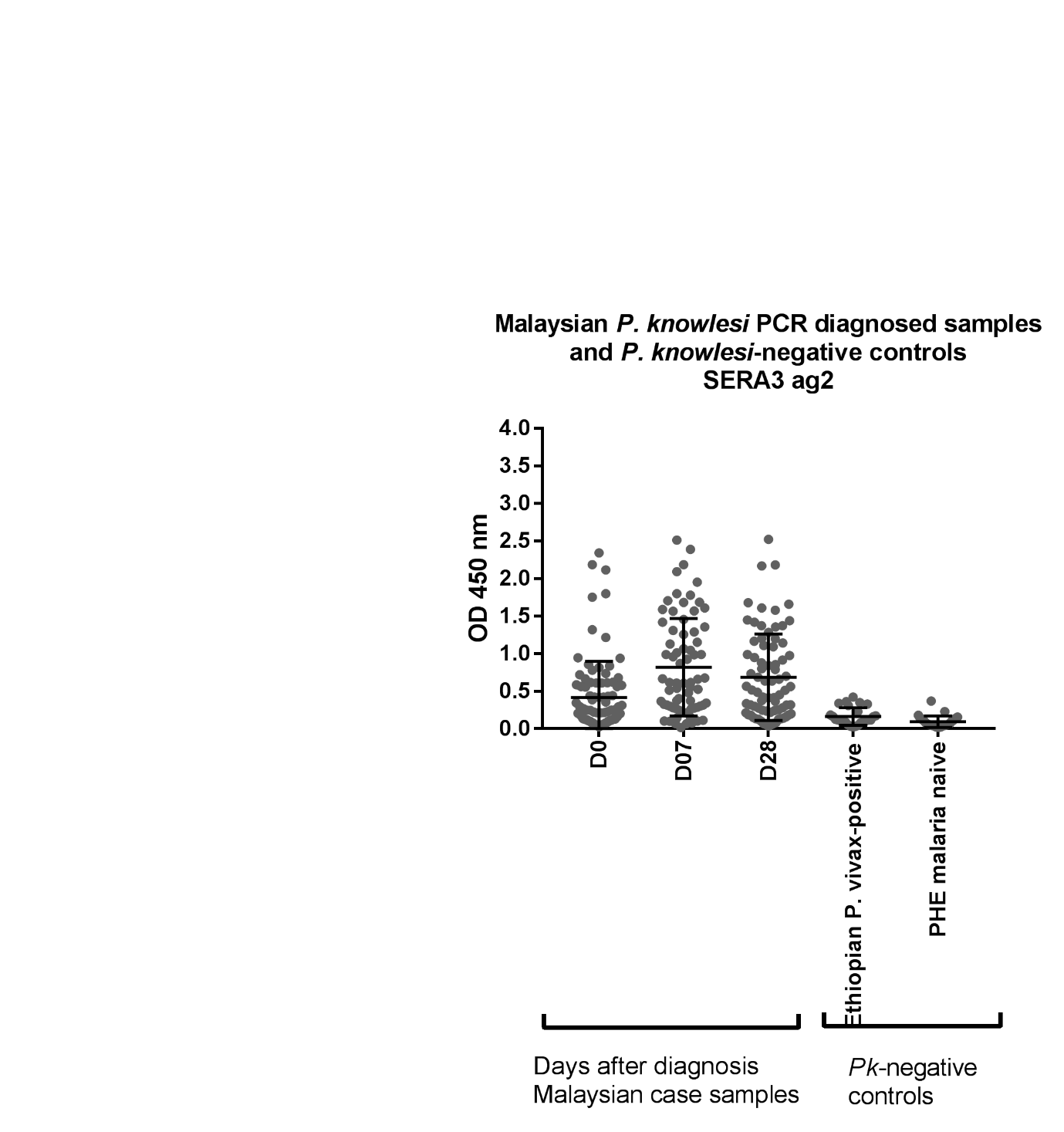
Supplementary Figure 4b


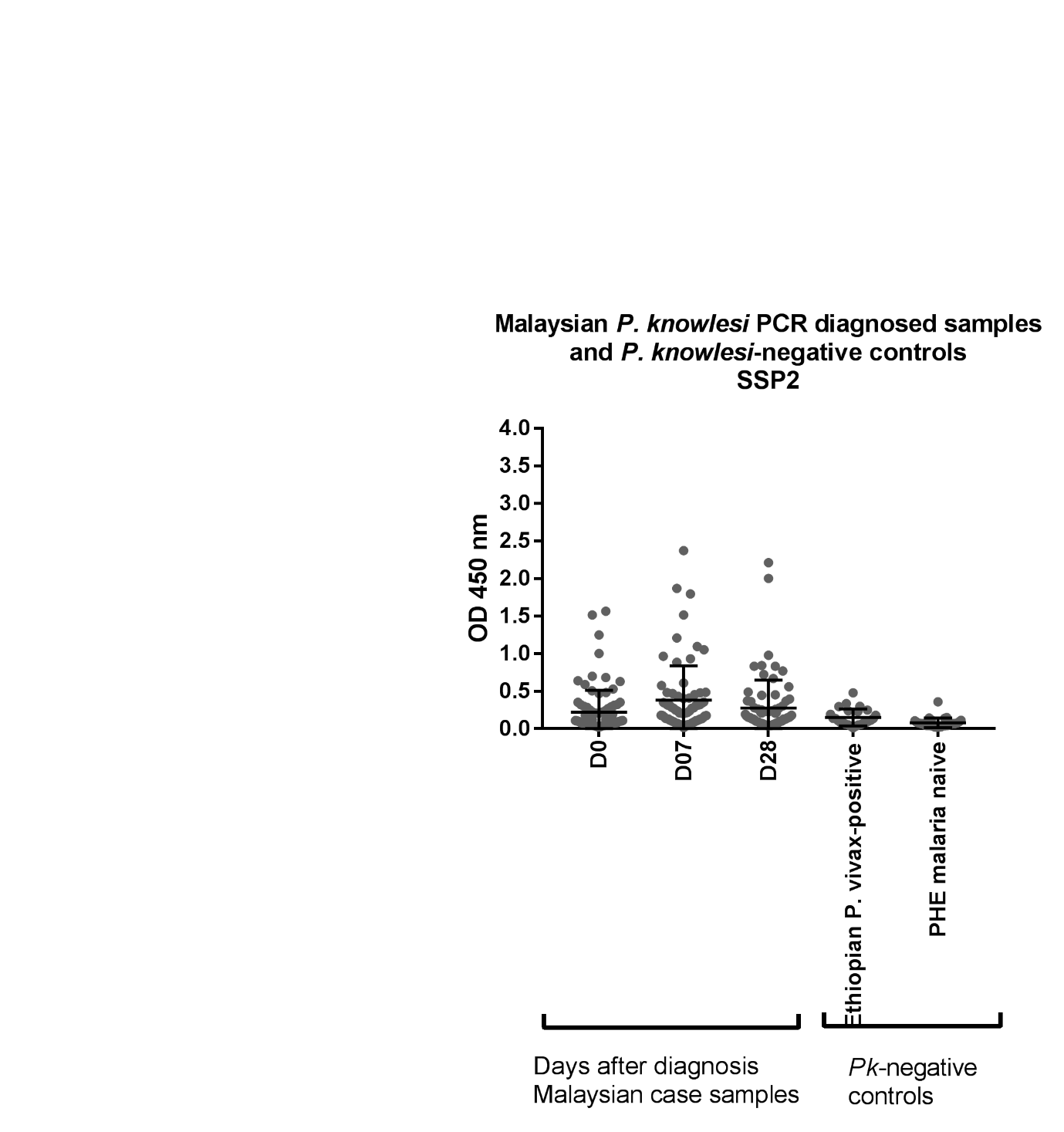
 Supplementary Figure 4c


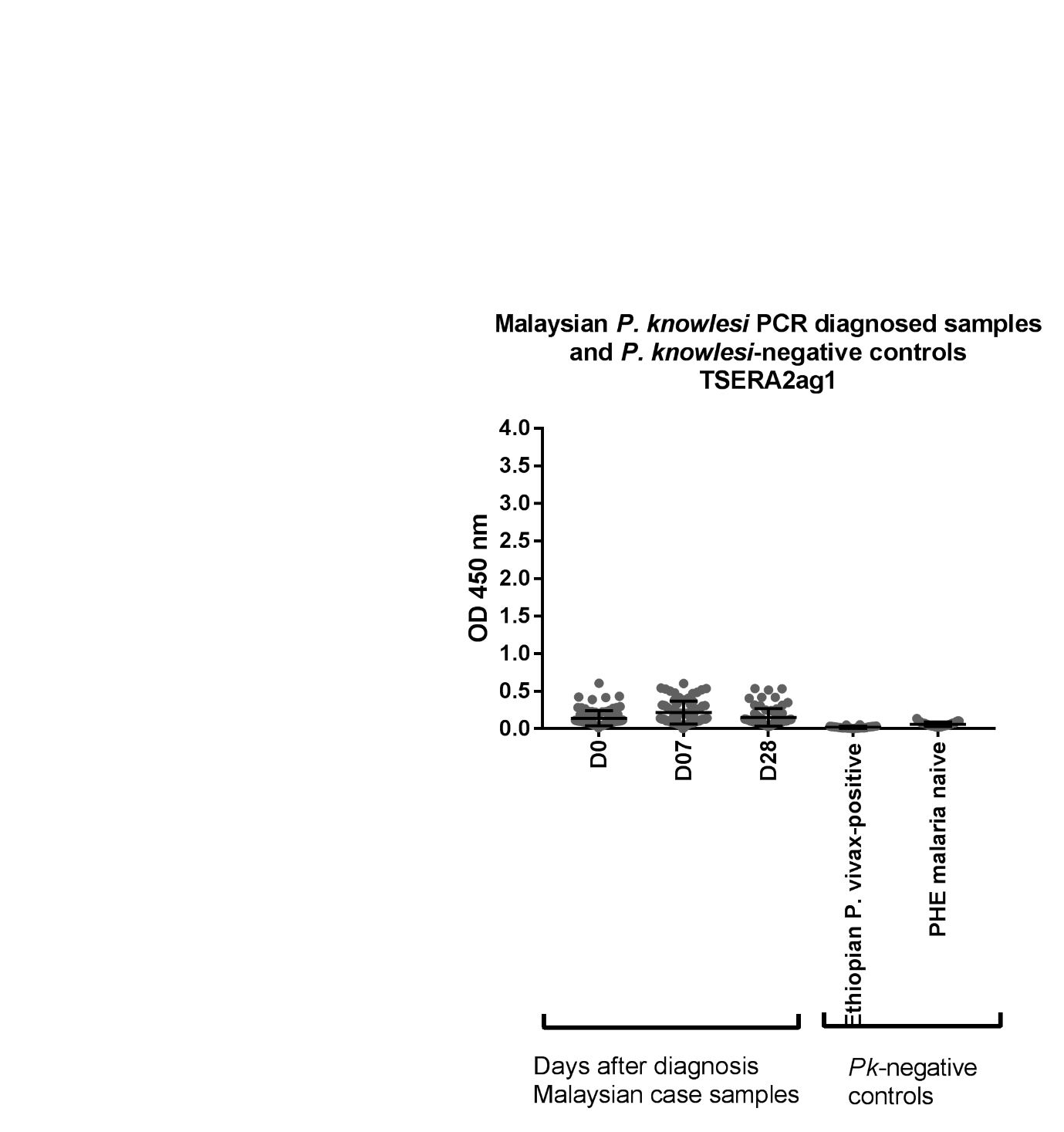
Supplementary Figure 4d
